# Gender inequalities in the disruption of long-term life satisfaction trajectories during the COVID-19 pandemic and the role of time use: evidence from a prospective cohort study

**DOI:** 10.1192/bjo.2024.817

**Published:** 2024-12-04

**Authors:** Darío Moreno-Agostino, Jenny Chanfreau, Gemma Knowles, Alina Pelikh, Jayati Das-Munshi, George B. Ploubidis

**Affiliations:** ESRC Centre for Society and Mental Health, King's College London, UK; and Centre for Longitudinal Studies, UCL Social Research Institute, University College London, UK; Centre for Longitudinal Studies, UCL Social Research Institute, University College London, UK; and Department of Sociology and Criminology, University of Sussex, UK; ESRC Centre for Society and Mental Health, King's College London, UK; and Department of Health Service and Population Research, Institute of Psychiatry, Psychology & Neuroscience, King's College London, UK; Centre for Longitudinal Studies, UCL Social Research Institute, University College London, UK; ESRC Centre for Society and Mental Health, King's College London, UK; Department of Psychological Medicine, Institute of Psychiatry, Psychology & Neuroscience, King's College London, UK; and South London and Maudsley NHS Foundation Trust, London, UK

**Keywords:** Epidemiology, longitudinal data, structural equation modelling, latent variable modelling, statistical methodology

## Abstract

**Background:**

The COVID-19 pandemic has disproportionately affected women's mental health. However, most evidence has focused on mental illbeing outcomes, and there is little evidence on the mechanisms underlying this unequal impact.

**Aims:**

To investigate gender differences in the long-term trajectories of life satisfaction, how these were affected during the pandemic and the role of time-use differences in explaining gender inequalities.

**Method:**

We used data from 6766 (56.2% women) members of the 1970 British Cohort Study (BCS70). Life satisfaction was prospectively assessed between the ages of 26 (1996) and 51 (2021) years, using a single question with responses ranging from 0 (lowest) to 10 (highest). We analysed life satisfaction trajectories with piecewise latent growth curve models, and investigated whether gender differences in the change in the life satisfaction trajectories with the pandemic were explained by self-reported time spent doing different paid and unpaid activities.

**Results:**

Women had consistently higher life satisfaction than men before the pandemic (Δ_intercept,unadjusted_ = 0.213, 95% CI 0.087–0.340; *P =* 0.001) and experienced a more accelerated decline with the pandemic onset (Δ_quad2,unadjusted_ = −0.018, 95% CI −0.026 to −0.011; *P* < 0.001). Time-use differences did not account for the more accelerated decrease in women's life satisfaction levels with the pandemic (Δ_quad2,adjusted_ = −0.016, 95% CI −0.031 to −0.001; *P* = 0.035).

**Conclusions:**

Our study shows pronounced gender inequalities in the impact of the pandemic on the long-term life satisfaction trajectories of adults in their 50s, with women losing their pre-pandemic advantage over men. Self-reported time-use differences did not account for these inequalities. More research is needed to tackle gender inequalities in population mental health.

There have been concerns that the COVID-19 pandemic has inequitably affected the mental health of different groups within the population.^1,2^ Certain groups, including women, have been disproportionately affected, with evidence suggesting that pre-existing inequalities widened with the pandemic onset.^3–5^ Multiple studies have tentatively proposed that gender inequalities in how people used their time may be partly responsible for this unequal impact, as women, on average, had a disproportionate burden of the additional caregiving responsibilities and household chores.^6–9^ Cross-sectional evidence has suggested that such a relationship may exist, with women spending more time than men doing these kinds of activities during the pandemic, leading to lower well-being levels.^10^ However, empirical evidence on whether such differences in time use may explain more negative changes in mental health typically experienced by women with the onset and course of the pandemic is lacking. Moreover, although there is evidence on the impact of the pandemic on the long-term trajectories of mental illbeing outcomes such as psychological distress,^3^ such evidence is not available for mental well-being outcomes such as life satisfaction. These outcomes do not simply represent the other pole of the same axis, as evidenced by studies showing that correlates of mental illbeing are not necessarily the same as correlates of mental well-being.^11,12^ It is possible to experience high levels of both distress and well-being, and the so-called ‘gender paradox’ is precisely an example of this. This paradox refers to the finding that, despite being generally exposed to more disadvantage and experiencing higher levels of mental illbeing, women often have higher mental well-being levels than men at the population level.^13–16^ However, recent evidence suggests that, during the pandemic, women may have lost that relative advantage in mental well-being,^17,18^ without clear evidence on the potential mechanisms leading to a larger decline in women.

The aims of this study are twofold. First, to examine differences across women and men in the impact of the pandemic in their long-term trajectories of life satisfaction. This can provide relevant insights on whether there was a continuation of pre-existing trends or an unexpected disruption, as noted for psychological distress.^3^ Second, to formally investigate whether differences in the way in which women and men spent their time during the pandemic could explain, at least partly, any observed disparities in the way in which life satisfaction changed with the onset and course of the pandemic across these two groups. This can help reveal how gender norms and societal expectations shape individuals’ experiences and well-being during times of crisis, and inform more gender-responsive policy responses to crises.

## Method

### Sample and procedure

We used data from the 1970 British Cohort Study (BCS70), a birth cohort following the lives of around 17 000 people born in Great Britain in a single week in 1970.^19^ Data on life satisfaction were collected at years 1996, 2000, 2004, 2012 and 2016 (corresponding to ages 26, 30, 34, 42 and 46 years). Cohort data was augmented with data from the COVID-19 survey,^20^ which collected data from the cohort members at three time points during the pandemic: May 2020 (during the first nationwide lockdown), September/October 2020 (during a period of eased restrictions) and February/March 2021 (during the third nationwide lockdown), corresponding to ages 50, 50.5 and 51 years, respectively. Cohort members that took part in any of these COVID-19 survey waves were included in this study, leading to a total of 6766 participants. Non-response weights were derived as part of the COVID-19 survey project, to restore the representativeness of the BCS70 to its target population (adults born in 1970 in Great Britain, alive and still residing in the UK during the pandemic), taking into account an extensive number of factors that have been found to be associated to non-response, in line with the Centre for Longitudinal Studies’ missing data strategy.^21^ All details on the derivation of these non-response weights and their effectiveness to restore sample representativeness and reduce bias are available in the COVID-19 Survey User Guide.^20^ All participants provided informed consent.

### Measures

Life satisfaction was assessed by a single question with an 11-point response scale, with 0 and 10 representing the lowest and highest satisfaction, respectively. The specific wording of the questions varied slightly across the survey waves (see Supplementary Appendix 1 available at https://doi.org/10.1192/bjo.2024.817).

As part of the first and second COVID-19 survey waves, participants were asked to report the number of hours they usually spent doing different activities in a typical weekday since the outbreak (i.e. their time use). In this study, we focused on the time spent doing paid work (working), volunteering/doing unpaid work (volunteering), home-schooling children (home-schooling), doing other interactive activities with children (caring:children), caring for someone other than a child (caring:other) and doing housework (housework). Based on the distribution of responses to these variables (see Supplementary Appendix 2), they were recoded into 0, ≤8 or >8 h (working); 0 or >0 h (volunteering, home-schooling, caring:children, caring:other); or 0, ≤1 or >1 h (housework). Only information on time spent working was collected during the third COVID-19 survey wave, which was transformed into the same scale as the data from the first two COVID-19 survey waves.

Complete data on gender assigned at birth was drawn from the birth sweep, as information on gender was not consistently available for participants. We believe that depicting any differences across groups as sex inequalities would risk essentialising them as attributable to inherent traits or biological characteristics rather than to the ways in which women and men are differentially socialised and oppressed throughout their life courses. Therefore, we refer to these as gender inequalities.

In the three COVID-19 survey waves, participants were asked to report their financial situation compared with before the pandemic outbreak (‘Much worse off’, ‘A little worse off’, ‘About the same’, ‘A little better off’ or ‘Much better off’), their work location (used to derive a variable capturing whether the person worked completely/partially from home or not) and their keyworker status (yes/no). We derived a variable capturing whether the person was living with dependent children or young people aged ≤16 years.^6^ These four measures were considered as key aspects that could confound the relationship between time use and life satisfaction.^10^

### Data analyses

#### Life-course trajectories of life satisfaction

To address the first aim of the study, we used a latent growth curve modelling (LGCM) approach,^22^ using observations from all data points from years 1996 to 2021. Models were unadjusted to avoid controlling for variables that may be on the pathway between gender and life satisfaction.^23^ We analysed the life satisfaction trajectories across men and women separately, using a model comparison strategy to identify the best-fitting functional form among a prespecified set of options based on the inspection of the descriptive data. An intercepts-only/no change LGCM was estimated as a baseline model. We then estimated and compared the fit across (a) polynomial trajectories (linear, quadratic and cubic change), (b) piecewise models with the knot at age 46 years to accommodate the change in the trajectory shape with the pandemic onset, and (c) a free/‘latent basis’ trajectory shape. All LGCM models were estimated with maximum likelihood with robust s.e. (MLR). Comparative fit of nested models was tested with the Satorra-Bentler scaled robust *χ*^2^ difference test,^24^ with a significant result indicating better fit of the most flexible model; whereas non-nested models were compared based on the information criteria (Bayesian information criterion and Akaike's information criterion), with lower information criteria values indicating better fit.

Once the optimal functional form was identified for each gender, a multiple group LGCM strategy was implemented in which increasing number of equality constraints across groups were specified to identify the most parsimonious version of the trajectories supported by the data. Then, equality of growth factors across genders was formally tested with Wald tests.

#### The role of time-use differences

To address the second aim of the study, we first introduced the time-use variables as predictors of changes in life satisfaction during the COVID-19 pandemic. We then further included financial situation, working from home, keyworker status and living with any dependent child or young person aged ≤16 years as potential confounders of the relationship between time use and life satisfaction, based on previous literature^10^ and data availability. The rationale behind these analyses is that, if time use could account for the gender gap in life satisfaction during the pandemic, the gender differences in the growth parameters representing the change during the pandemic would be attenuated. As an additional exploratory analysis, gender differences in the relationship between time spent in different activities and life satisfaction levels at each of the COVID-19 survey waves were tested with Wald tests. Additional details on the analytical approach are provided in Supplementary Appendix 3.

#### Missing data

Inverse probability weighting was used to account for differential unit missingness and to restore the representativeness of the sample to the reference population, using the most recently available non-response weights for each individual.^20^ Item missingness was accounted for by using full information maximum likelihood.^25^ Both strategies were used simultaneously to render the missing-at-random assumption more tenable.

#### Sensitivity analyses

Information on life satisfaction was collected across all waves, using pen-and-paper (age 26 years sweep) or computer (ages 30–51 years) self-reported questionnaires. A small portion (3.2%) of the participants in the last COVID-19 survey wave (February/March 2021) were interviewed by telephone to boost response. Since this subgroup of participants were allocated into this data collection mode not at random but based on their non-response to the online survey, this resulted in differences in some of the collected variables (including life satisfaction) potentially attributable to data collection mode.^20^ Since mode effects in life satisfaction measures have been long reported in the literature,^26,27^ we estimated the final unconditional and adjusted LGCMs including the interview mode as a covariate.

Since information on time use at the last time-point was limited, and to further ensure the temporal ordering of the variables in the analyses, we estimated an additional set of models in which the time-use variables and the confounders had a lagged (rather than concurrent) effect on the next life satisfaction assessment.

As a result of the review process, we conducted an additional set of sensitivity analyses where high self-reported values of hours spent doing paid work (≥20 h/day) were treated as missing data.

Data management was carried out in Stata for Windows version MP 17.^28^ LGCM models were estimated in Mplus for Windows version 8.^29^ BCS70 deidentified data used for this study is available at the UK Data Service.^30^ Example code of the unadjusted and adjusted multiple groups LGCMs is available on the Open Science Framework: https://osf.io/7esnw/.

The authors assert that all procedures contributing to this work comply with the ethical standards of the relevant national and institutional committees on human experimentation and with the Helsinki Declaration of 1975, as revised in 2013. All procedures involving human patients were approved by the National Health Service (NHS) Research Ethics Committee. All participants provided verbal informed consent.

## Results

Of the 6766 participants who were alive and resided in the UK during the COVID-19 pandemic, 3799 (56.2%) were women. A total of 42 804 observations were included, with a mean of 6.3 and a median of 7 observations per individual (interquartile range: 5–8). The mean and dispersion of life satisfaction levels over time between the ages of 26 and 51 years (Table 1) was higher around midlife (between ages 34 and 42 years), seemingly dropping at an increasingly accelerated pace after that, coinciding with the pandemic onset. Mean life satisfaction levels were higher among women than men before the pandemic onset (at age 50 years), but this reversed during the pandemic. Visual depictions of the individual (unweighted) and mean (weighted) life satisfaction levels by gender are provided in Supplementary Appendices 4 and 5, respectively. A larger proportion of women than men reported spending time volunteering, caring for others and doing >1 h of housework; women were more likely to be keyworkers and less likely to work from home. A larger proportion of men than women reported working >8 h, spending time taking care of children (beyond home-schooling), working from home, being in a better financial situation than before the outbreak and living with dependent children or young people. During the first lockdown (age 50 years), a larger proportion of women than men reported spending time home-schooling children, but this was reversed before the reintroduction of lockdown measures in Autumn 2020 (age 50.5 years). Detailed information on the variables on time use, financial situation, working from home, keyworker status and living with dependent children or young people aged ≤16 years, collected during the COVID-19 pandemic, is provided in Supplementary Appendix 6.

**Table 1:** Mean and dispersion of life satisfaction levels over time

	Men			Women			Overall		
Age (in years)/year	Total number of observations	Mean	s.d.	Total number of observations	Mean	s.d.	Total number of observations	Mean	s.d.
26/1996	2117	7.21	1.81	2961	7.36	1.85	5078	7.30	1.83
30/2000	2522	7.35	1.65	3374	7.51	1.79	5896	7.44	1.73
34/2004	2376	7.49	1.59	3188	7.57	1.73	5564	7.54	1.67
42/2012	2628	7.46	1.80	3414	7.56	1.87	6042	7.52	1.84
46/2016	2586	7.37	1.75	3289	7.53	1.81	5875	7.46	1.79
50/2020 (May)^a^	1599	7.25	1.90	2309	7.22	1.95	3908	7.23	1.93
50.5/2020 (September/October)^a^	2102	7.17	1.93	2895	7.09	1.95	4997	7.13	1.94
51/2021 (February/March)^a^	2312	6.93	1.96	3132	6.70	2.05	5444	6.80	2.02

Unweighted results. Life satisfaction measure ranges from 0 (minimum) to 10 (maximum).

a. Data collected as part of the COVID-19 survey waves.

### Life-course trajectories of life satisfaction

**Fig. 1:**
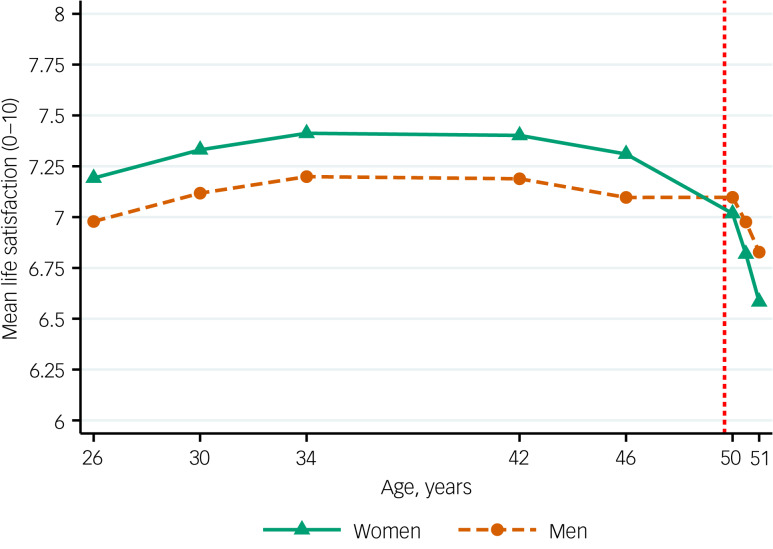
Unconditional life satisfaction trajectories across men and women (estimated, model-based means). The vertical red dashed line represents the COVID-19 pandemic onset.

A piecewise model with two quadratic segments had the best fit in both women and men, as evidenced by the model comparison strategy implemented to select the optimal functional form (fit indices of these models are provided in Supplementary Appendix 7). The multiple group LCGM approach implemented to identify the most parsimonious model supported the inclusion of multiple equality constraints across genders in the long-term trajectories of life satisfaction (fit indices of these models are provided in Supplementary Appendix 8). However, significant differences across groups were found in the intercepts – or starting points, located at age 26 years – being 0.213 (95% CI 0.087–0.340; *P* = 0.001) points higher among women; and in the quadratic change of the second segment – the accelerated decrease with the pandemic – being −0.018 (95% CI −0.026 to −0.011; *P* < 0.001) more accelerated among women (Table 2). A visual representation of the resulting life satisfaction trajectories is provided in Fig. 1.

**Table 2:** Results from the unconditional multiple group latent growth curve model (*N* = 6766)

	Women			Men		
	Point estimate	95% CI, lower bound	95% CI, upper bound	Point estimate	95% CI, lower bound	95% CI, upper bound
Means						
Intercept	7.192	7.099	7.285	6.979	6.864	7.093
Linear change, first segment	*0*.*042*	*0*.*026*	*0*.*058*	*0*.*042*	*0*.*026*	*0*.*058*
Quadratic change, first segment	*−0*.*002*	*−0*.*003*	*−0*.*001*	*−0*.*002*	*−0*.*003*	*−0*.*001*
Linear change, second segment	*0*.*216*	*0*.*119*	*0*.*312*	*0*.*216*	*0*.*119*	*0*.*312*
Quadratic change, second segment	−0.072	−0.092	−0.052	−0.054	−0.075	−0.033
Variances						
Intercept	*1*.*697*	*1*.*464*	*1*.*930*	*1*.*697*	*1*.*464*	*1*.*930*
Linear change, first segment	*0*.*115*	*0*.*085*	*0*.*144*	*0*.*115*	*0*.*085*	*0*.*144*
Quadratic change, first segment	*0*.*000*			*0*.*000*		
Linear change, second segment	*1*.*887*	*1*.*512*	*2*.*261*	*1*.*887*	*1*.*512*	*2*.*261*
Quadratic change, second segment	*0*.*000*			*0*.*000*		
Standardised covariances (correlations)						
Intercept × linear change, first segment	−0.347	−0.472	−0.221	−0.321	−0.451	−0.191
Intercept × linear change, second segment	−0.093	−0.215	0.029	−0.107	−0.270	0.056
Linear change first × second segment	−0.128	−0.282	0.026	−0.132	−0.328	0.063
Standardised residual variances (age in years)						
Age 26	0.557	0.500	0.613	0.551	0.489	0.613
Age 30	0.564	0.518	0.611	0.516	0.456	0.576
Age 34	0.548	0.502	0.593	0.481	0.419	0.543
Age 42	0.544	0.483	0.605	0.508	0.452	0.563
Age 46	0.399	0.325	0.473	0.383	0.300	0.465
Age 50	0.414	0.342	0.487	0.271	0.221	0.322
Age 50.5	0.299	0.257	0.341	0.283	0.222	0.345
Age 51	0.338	0.287	0.390	0.275	0.222	0.329
Between-groups difference in coefficients^a^	Point estimate	95% CI, lower bound	95% CI, upper bound	*P*-value		
Mean intercept	0.213	0.087	0.340	0.001		
Mean quadratic change, second segment	−0.018	−0.026	−0.011	<0.001		

Models estimated with non-response weights and full information maximum likelihood under maximum likelihood with robust s.e. estimation. Estimates in italics are constrained to be equal across groups. All within-group estimates are significant with *P* < 0.001, except the correlations involving the linear change in the second segment.

a. Between-groups difference in coefficients tested with Wald tests under the null hypothesis of estimate_women_ – estimate_men_ = 0.

The inclusion of the time-use variables resulted in a small attenuation of the differences across women and men in the accelerated change with the pandemic (represented by the quadratic term of the second segment of the piecewise latent growth model) and a large widening of the confidence interval (Δ_quad2_ = −0.016, 95% CI −0.031 to −0.001; *P* = 0.035). Adding the covariates representing the financial situation, working from home and keyworker status, and the presence of dependent children or young people aged ≤16 years in the household did not substantially change the differences across men and women in the accelerated decline in life satisfaction during the pandemic (Δ_quad2_ = −0.022, 95% CI −0.038 to −0.006; *P* = 0.008) (Table 3).

**Table 3:** Results from the adjusted multiple group latent growth curve models (*N* = 6766)

	Adjusted for time-use variables						Fully adjusted^a^					
	Women			Men			Women			Men		
	Point estimate	95% CI, lower bound	95% CI, upper bound	Point estimate	95% CI, lower bound	95% CI, upper bound	Point estimate	95% CI, lower bound	95% CI, upper bound	Point estimate	95% CI, lower bound	95% CI, upper bound
Means												
Intercept	7.188	7.093	7.283	6.970	6.852	7.087	7.185	7.090	7.280	6.966	6.848	7.084
Linear change, first segment	*0*.*042*	*0*.*026*	*0*.*057*	*0*.*042*	*0*.*026*	*0*.*057*	*0*.*042*	*0*.*027*	*0*.*058*	*0*.*042*	*0*.*027*	*0*.*058*
Quadratic change, first segment	*−0*.*002*	*−0*.*002*	*−0*.*001*	*−0*.*002*	*−0*.*002*	*−0*.*001*	*−0*.*002*	*−0*.*002*	*−0*.*001*	*−0*.*002*	*−0*.*002*	*−0*.*001*
Linear change, second segment	*0*.*266*	*−0*.*091*	*0*.*622*	*0*.*266*	*−0*.*091*	*0*.*622*	*0*.*265*	*−0*.*107*	*0*.*637*	*0*.*265*	*−0*.*107*	*0*.*637*
Quadratic change, second segment	−0.089	−0.162	−0.017	−0.073	−0.152	0.006	−0.090	−0.166	−0.014	−0.068	−0.151	0.014
Variances												
Intercept	*1*.*716*	*1*.*479*	*1*.*954*	*1*.*716*	*1*.*479*	*1*.*954*	*1*.*720*	*1*.*481*	*1*.*960*	*1*.*720*	*1*.*481*	*1*.*960*
Linear change, first segment	*0*.*115*	*0*.*086*	*0*.*145*	*0*.*115*	*0*.*086*	*0*.*145*	*0*.*115*	*0*.*085*	*0*.*144*	*0*.*115*	*0*.*085*	*0*.*144*
Quadratic change, first segment	*0*			*0*			*0*			*0*		
Linear change, second segment	*1*.*874*	*1*.*504*	*2*.*244*	*1*.*874*	*1*.*504*	*2*.*244*	*1*.*821*	*1*.*459*	*2*.*184*	*1*.*821*	*1*.*459*	*2*.*184*
Quadratic change, second segment	*0*			*0*			*0*			*0*		
Standardised covariances (correlations)												
Intercept × linear change, first segment	−0.351	−0.476	−0.226	−0.318	−0.449	−0.188	−0.350	−0.476	−0.224	−0.316	−0.446	−0.185
Intercept × linear change, second segment	−0.098	−0.220	0.024	−0.129	−0.291	0.033	−0.119	−0.242	0.004	−0.147	−0.313	0.018
Linear change first × second segment	−0.144	−0.297	0.009	−0.133	−0.325	0.059	−0.148	−0.301	0.006	−0.124	−0.317	0.070
Standardised residual variances (age in years)												
Age 26	0.554	0.498	0.610	0.547	0.487	0.607	0.554	0.498	0.610	0.547	0.488	0.606
Age 30	0.561	0.515	0.608	0.512	0.453	0.572	0.561	0.515	0.608	0.511	0.451	0.571
Age 34	0.545	0.500	0.591	0.477	0.417	0.537	0.543	0.497	0.589	0.476	0.416	0.536
Age 42	0.543	0.482	0.603	0.502	0.448	0.557	0.543	0.482	0.603	0.500	0.445	0.554
Age 46	0.398	0.325	0.471	0.378	0.300	0.457	0.400	0.327	0.472	0.380	0.303	0.457
Age 50	0.418	0.348	0.487	0.268	0.220	0.315	0.416	0.359	0.474	0.269	0.221	0.317
Age 50.5	0.281	0.240	0.322	0.292	0.234	0.350	0.285	0.245	0.324	0.288	0.231	0.346
Age 51	0.339	0.289	0.389	0.260	0.210	0.311	0.326	0.280	0.373	0.254	0.205	0.303
Effects on life satisfaction at age 50 years												
Working 1–8 h	0.200	−0.041	0.442	0.154	−0.059	0.368	0.166	−0.130	0.462	0.123	−0.124	0.371
Working >8 h	0.217	−0.086	0.521	−0.066	−0.300	0.168	0.193	−0.163	0.549	−0.085	−0.349	0.179
Volunteering ≥1 h	−0.174	−0.432	0.084	0.091	−0.246	0.429	−0.160	−0.410	0.091	0.081	−0.249	0.411
Home-schooling ≥1 h	0.148	−0.102	0.398	−0.036	−0.249	0.178	0.247	−0.050	0.543	−0.069	−0.298	0.161
Caring for children ≥1 h	−0.158	−0.418	0.102	−0.125	−0.310	0.060	−0.069	−0.335	0.197	−0.121	−0.329	0.087
Caring for others ≥1 h	0.246	−0.035	0.527	0.211	−0.043	0.465	0.217	−0.055	0.490	0.219	−0.037	0.474
Doing housework 1 h	−0.127	−0.473	0.219	0.118	−0.088	0.323	−0.166	−0.532	0.199	0.117	−0.090	0.324
Doing housework ≥2 h	−0.172	−0.545	0.201	−0.002	−0.223	0.218	−0.218	−0.624	0.189	−0.008	−0.228	0.212
Financial situation							0.106	−0.077	0.288	0.139	0.043	0.235
Working from home							0.181	−0.016	0.377	0.059	−0.153	0.272
Keyworker status							0.125	−0.102	0.352	−0.018	−0.221	0.184
Dependent CYP in the household							−0.080	−0.374	0.214	−0.065	−0.289	0.158
Effects on life satisfaction at age 50.5 years												
Working 1–8 h	0.489	0.257	0.721	−0.126	−0.420	0.168	0.338	0.105	0.571	−0.197	−0.497	0.103
Working >8 h	0.389	0.135	0.643	−0.289	−0.623	0.046	0.251	−0.005	0.508	−0.309	−0.640	0.022
Volunteering ≥1 h	0.101	−0.106	0.308	0.268	0.010	0.527	0.096	−0.110	0.302	0.228	−0.030	0.487
Home-schooling ≥1 h	−0.153	−0.420	0.113	−0.087	−0.422	0.249	−0.190	−0.464	0.084	−0.144	−0.500	0.212
Caring for children ≥1 h	0.133	−0.023	0.289	0.131	−0.069	0.331	0.120	−0.052	0.293	0.100	−0.160	0.360
Caring for others ≥1 h	−0.190	−0.389	0.008	0.236	−0.097	0.570	−0.200	−0.403	0.002	0.285	−0.059	0.629
Doing housework 1 h	−0.176	−0.456	0.104	0.349	0.078	0.620	−0.203	−0.483	0.077	0.352	0.075	0.629
Doing housework ≥2 h	−0.150	−0.422	0.121	0.230	−0.104	0.564	−0.160	−0.432	0.112	0.249	−0.088	0.586
Financial situation							0.115	0.019	0.212	0.148	0.041	0.255
Working from home							0.376	0.201	0.551	0.231	0.014	0.448
Keyworker status							0.171	−0.033	0.374	−0.255	−0.493	−0.017
Dependent CYP in the household							0.007	−0.255	0.268	−0.038	−0.315	0.238
Effects on life satisfaction at age 51 years												
Working 1–8 h	0.245	0.005	0.486	0.282	−0.053	0.618	0.286	−0.022	0.595	0.182	−0.190	0.554
Working >8 h	0.125	−0.189	0.439	0.296	−0.043	0.635	0.185	−0.191	0.561	0.204	−0.171	0.578
Financial situation							0.225	0.123	0.326	0.181	0.083	0.278
Working from home							0.060	−0.164	0.284	−0.008	−0.264	0.247
Keyworker status							−0.050	−0.300	0.201	0.068	−0.168	0.304
Dependent CYP in the household							0.100	−0.209	0.409	−0.138	−0.424	0.148
Between-groups difference in coefficients^b^	Point estimate	95% CI, lower bound	95% CI, upper bound	*P*-value			Point estimate	95% CI, lower bound	95% CI, upper bound	*P*-value		
Mean intercept	0.218	0.066	3.308	0.001			0.219	0.089	0.348	0.001		
Mean quadratic change, second segment	−0.016	−0.031	−0.001	0.035			−0.022	−0.038	−0.006	0.008		

Models estimated with non-response weights and full information maximum likelihood under maximum likelihood with robust s.e. estimation. Estimates in italics are constrained to be equal across groups. Effects of time-use variables are estimated with no time spent in that activity as a reference category. CYP, children or young people.

a. Adjusted for financial situation, working from home and keyworker status, and presence in the household of dependent children or young people aged up to 16 years.

b. Between-groups difference in coefficients tested with Wald tests under the null hypothesis of estimate_women_ – estimate_men_ = 0.

Significant gender differences in the relationship between four time-use variables and life satisfaction were found in September/October 2020, a period without lockdown restrictions: working (doing paid work) was positively related to life satisfaction in women, but negatively in men (Δ_working1−8h_ = 0.535, 95% CI 0.156–0.914, *P* = 0.006; Δ_working > 8h_ = 0.560, 95% CI 0.143–0.978, *P* = 0.009); however, spending time caring for others (not children, Δ_caring1h_ = −0.486, 95% CI −0.884 to −0.087, *P* = 0.017) and doing housework (Δ_housework1h_ = −0.555, 95% CI −0.956 to −0.155, *P* = 0.007) was negatively related to life satisfaction in women, but positively in men. Results from the Wald test conducted on the fully adjusted models are provided in Supplementary Appendix 9.

Results (and, chiefly, the difference across women and men in the accelerated change in life satisfaction with the pandemic after accounting for the time-use variables and covariates) were robust to the adjustment for potential mode effects (Δ_quad2_ = −0.027, 95% CI −0.044 to −0.010, *P* = 0.001) (Supplementary Appendix 10), to the inclusion of lagged effects from the time-use variables and the confounders (Δ_quad2_ = −0.025, 95% CI −0.039 to −0.011, *P* < 0.001) (Supplementary Appendix 11), and to treating high self-reported values of hours spent doing paid work as missing data (Δ_quad2_ = −0.027, 95% CI −0.044 to −0.010, *P* = 0.002) (Supplementary Appendix 12).

## Discussion

Our study shows, for the first time, that the long-term trajectories of life satisfaction of British adults in their 50s significantly changed with the COVID-19 pandemic, reaching their lowest levels in up to 25 years of follow-up data. This decline was particularly salient among women, who lost their pre-pandemic advantage over men reversing the pre-existing gender gap in life satisfaction, and our study suggests that self-reported differences in time use did not explain this differential impact. Although this pre-pandemic advantage may seem small, its size (around 0.213 points higher life satisfaction in women than in men) is similar to the effect of not having health problems preventing engagement in activities that people of similar age (people aged 44–57 years) can normally do, as reported in a population-based study with data from multiple countries.^31^ Women not only lost that advantage, but their levels kept declining at a more rapid pace than men's. Unlike what has been suggested in previous studies,^17^ our long-term longitudinal analysis does not suggest that this gendered change had started to occur before the pandemic (or at least not by 2016, the most recent pre-pandemic assessment). Our study aligns with recent research showing the disproportionate impact of the pandemic on women's mental health outcomes in the UK^1,3–5,32^ and complements existing literature on mental illbeing outcomes such as psychological distress,^3^ by placing that differential impact in the wider context of pre-existing long-term trajectories of life satisfaction. Unlike the evidence on mental illbeing, suggesting the widening of pre-existing inequalities,^3^ the present study focuses on a mental well-being outcome and suggests that the pandemic resulted in the emergence of a new, non-pre-existent inequality, with women born in 1970 losing their pre-pandemic advantage over men in life satisfaction. Although this can be interpreted as a resolution for a ‘gender paradox’,^17^ with women now having both lower mental well-being and higher mental illbeing outcomes, it raises the important question of what potential mechanisms are driving this inequality.

In this regard, we engaged with that important question by investigating whether differences in how men and women used their time during the pandemic could account for at least part of this gap, but found inconclusive evidence. Our results are not consistent with previous studies suggesting that time-use differences might explain the gender gap in mental health.^3,32^ Women's higher engagement in activities typically associated with higher mental illbeing and lower mental well-being, such as doing housework and caring for others, did not account for this disparity. A potential explanation for this is that it is not the absolute time spent in the activity that may drive the gender gap in mental health, but the proportion of it that women do.^33^ Additionally, society not only holds women accountable for these unpaid responsibilities, but women also tend to be responsible for coordinating and monitoring these activities even when not involved in their execution, to a greater extent than men. This so-called ‘mental load’ has been proposed to have a ubiquitous toll on their cognitive and affective state as coordination and monitoring transcend time and place.^34^ Therefore, it is likely that this invisible, boundaryless labour plays a role in the observed life satisfaction inequalities during the pandemic. It is also possible that this ‘mental load’ went beyond the personal household and intensified during the pandemic, since adults in this age group are expected to care for their offspring and older parents in what has been called the ‘sandwich generation’,^35^ potentially driving further gender inequalities. We also found that, during a period of reduced restrictions (September/October 2020), paid work, care work and housework had different effects on life satisfaction for men and women. It may be that women, who were less likely to remain in the labour market,^36^ could derive more satisfaction from engaging in activities (i.e. working) that had been more restricted in the initial stages of the pandemic. These results raise questions about whether there are systematic differences in the kind of activities that men and women report under general categories such as ‘doing housework’, which, in the administered questionnaire, comprised activities such as do-it-yourself (DIY) activities and household management chores, which may be related to higher and lower well-being, respectively. Overall, our study suggests that self-reported time-use differences did not explain the differential impact of the pandemic on women's life satisfaction, even after accounting for potentially confounding factors such as the financial, occupational and household composition situation. Future research on other potential mechanisms, including the ability to socialise and perceive/receive social support,^37,38^ the exposure to intimate partner violence and worries about personal safety,^39^ the menopausal transition^40^ and the ‘mental load’,^34^ is warranted to provide a deeper understanding of the mechanisms of the differential impact.

### Strengths and limitations

This study has several strengths and limitations. Unlike other studies conducted on convenience samples, we used data from a nationally representative cohort of people born in Great Britain in 1970, with data on the life satisfaction of the same individuals prospectively collected over 25 years, including during the COVID-19 pandemic. We accounted for differences in non-response by using appropriate methods to restore representativeness to the target population of adults alive and residing in the UK during the pandemic. By adopting a life-course perspective, this study is the first to offer evidence on the impact of the pandemic on long-term life satisfaction trajectories in British adults, explicitly exploring potential mechanisms contributing to the differences found across women and men.

However, these findings must be interpreted with caution because of several limitations. First, although the response options were nearly identical, slight variations in the wording of the life satisfaction questions over time may have introduced measurement error and influenced observed changes. Because of the single-item nature of the outcome, we could not empirically test its longitudinal measurement invariance or the extent of measurement error. However, our findings align with other sources,^17,18^ including cross-sectional evidence showing similar trends before and during the COVID-19 pandemic by gender in the UK, consistently using the same wording as that used in our study during the pandemic.^18^ Second, there are several limitations regarding the measurement and operationalisation of time use. The reliance on questionnaire measures rather than diary ones may have increased recall bias and social desirability effects, and previous research in the UK has suggested that questionnaire-based estimates may underestimate the gender gap in housework participation.^41^ The distribution and restricted range of many of the time-use variables may have also affected the results. We created categorical variables based on these distributions, highlighting the differences between no engagement and some or more than some engagement, but it is possible that different categorisations may have rendered different results. Additionally, as mentioned above, the broad categorisation of time-use activities hinders a more precise assessment of the specific activities encompassed in these reports. Furthermore, we could not adjust for differences in time use at the final data collection point, because of missing data in the corresponding survey wave. To mitigate this limitation, we included information on time spent working and the financial, occupational and household composition covariates, and also explored the potential lagged effects of the time-use variables, but the gender gaps in the life satisfaction changes persisted. Lack of time-use data before the pandemic meant that we were not able to analyse whether changes in the way in which people spent their time before and during the pandemic (and possibly differences across women and men in those changes) led to changes to their individual life satisfaction trajectories over time. Our study covers data up to the first quarter of 2021, and the gender gap may have evolved subsequently, with repeated cross-sectional data suggesting that women's and men's life satisfaction levels may have converged by the second half of 2021.^17,18^ Future research using higher-resolution longitudinal data extending beyond the first year of the pandemic, along with less biased data collection methods such as ecological momentary assessments or digital trackers, may yield additional insights into the role of time use in shaping the gender gap in the mental health of the same individuals. Although we focused on gender inequalities, the experiences of socialisation and oppression that lead to these are not homogeneous across other social identities and positions (e.g. ethnicity or sexual orientation), in line with intersectionality theories.^42,43^ Future research may explore such intersectional inequalities from a longitudinal perspective. Although our study's results may be generalisable to adults born in Great Britain in 1970 and residing in the UK, caution should be exercised when extending these findings to other segments of the UK population, such as migrants or different generations. For instance, younger generations may have been more likely to have younger children at home,^6^ with the impact of home-schooling and taking care of them being substantially different, potentially resulting in different gender inequalities. Finally, similar caution needs to be exercised when generalising these findings to other populations with different demographic, cultural and political characteristics.

In conclusion, this study reveals a substantial decline in life satisfaction among adults in their 50s during the first year of the COVID-19 pandemic in the UK. These levels reached the lowest point observed across 25 years of follow-up data. Notably, the decline was most pronounced among women, who transitioned from having higher life satisfaction than men during most of their adult lives, to experiencing an even more rapid decline with the pandemic's onset. Self-reported differences in time use did not account for the emergence of this new gender gap in life satisfaction. These results underscore the importance of ongoing monitoring of mental well-being in the population, especially as additional challenges (such as the cost-of-living crisis and underfunding of health, mental health, social care and education services) continue to affect mental illbeing and well-being. It is crucial to prioritise the most disadvantaged individuals in these efforts. Further research is needed to understand the mechanisms driving the substantial decline in life satisfaction at the population level, particularly the more accelerated decline observed among women. Such insights could inform the development of policies aimed at safeguarding and enhancing mental health during times of crisis.

## Supporting information

Moreno-Agostino et al. supplementary material Moreno-Agostino et al. supplementary material

## Data Availability

The data that support the findings of this study are openly available in the UK Data Service at http://doi.org/10.5255/UKDA-Series-200001.^30^
