# Supplementary material for: Gender inequalities in the disruption of long-term life satisfaction trajectories during the COVID-19 pandemic and the role of time use: evidence from a prospective cohort study
Source: BJPsych Open. 2024 Dec 4;10(6):e217. doi: 10.1192/bjo.2024.817 (PMC11698164; doi:10.1192/bjo.2024.817)
Supplement: Moreno-Agostino et al. supplementary material [file S2056472424008172sup001.docx]

Gender inequalities in the disruption of long-term life satisfaction trajectories
during the COVID-19 pandemic and the role of time use:
evidence from a prospective cohort study
Supplementary Material

Table of Contents

[Appendix S1. Life satisfaction question wording and mode of administration. 2](#_Toc178839591)

[Appendix S2. Distribution of responses to financial situation and time use variables. 3](#_Toc178839592)

[Appendix S3. Additional details on the rationale for the analytical approach. 7](#_Toc178839593)

[Appendix S4. Observed (unweighted) life satisfaction levels across a random (n=200) subset of cohort members over time. 9](#_Toc178839594)

[Appendix S5. Mean (weighted) observed life satisfaction levels over time. 10](#_Toc178839595)

[Appendix S6. Detailed information on the time-specific covariates (time use, financial situation, working from home, keyworker status, and dependent children or young people in the household) during the COVID-19 pandemic. 11](#_Toc178839596)

[Appendix S7. Fit indices for the latent growth curve models estimated to identify the optimal functional form in the overall, women, and men samples. 13](#_Toc178839597)

[Appendix S8. Fit indices for the multiple groups latent growth curve models estimated to identify the most parsimonious growth model across groups. 15](#_Toc178839598)

[Appendix S9. Results from the Wald tests analysing the difference in the relationship between time-use variables and life satisfaction across women and men, based on fully adjusted multiple group latent growth curve models (n=6,766). 17](#_Toc178839599)

[Appendix S10. Results from the multiple group latent growth curve models adjusted for interview mode (n=6,766). 18](#_Toc178839600)

[Appendix S11. Results from the multiple group latent growth curve models including lagged effects of the time-specific variables (n=6,766). 20](#_Toc178839601)

[Appendix S12. Results from the multiple group latent growth curve models including treating high self-reported values of hours spent doing paid work (≥20h/day) as missing data (n=6,766). 22](#_Toc178839602)

##

## Appendix S1. Life satisfaction question wording and mode of administration.

| Age / year | Life satisfaction question wording | Mode of administration |
| --- | --- | --- |
| 26 / 1996 | Here is a scale from 0 to 10. On it, “0” means that you are completely dissatisfied and “10” means that you are completely satisfied. Please tick the box with the number above it which shows how dissatisfied or satisfied you are about the way your life has turned out so far. | Pen-and-paper self-report questionnaire |
| 30 / 2000 | Here is a scale from 0-10 where ‘0’ means that you are completely dissatisfied and ‘10’ means that you are completely satisfied. Please enter the number which corresponds with how satisfied or dissatisfied you are about the way your life has turned out so far. | CASI |
| 34 / 2004 | Here is a scale from 0-10 where ‘0’ means that you are completely dissatisfied and ‘10’ means that you are completely satisfied. Please enter the number which corresponds with how satisfied or dissatisfied you are with the way life has turned out so far. | CASI |
| 42 / 2012 | Here is a scale from 0-10 where ‘0’ means that you are completely dissatisfied and ‘10’ means that you are completely satisfied. Please select the number which corresponds with how satisfied or dissatisfied you are with the way life has turned out so far. | CASI |
| 46 / 2016 | Here is a scale from 0-10 where ‘0’ means that you are completely dissatisfied and ‘10’ means that you are completely satisfied. Please select the number which corresponds with how satisfied or dissatisfied you are with the way life has turned out so far. | CASI |
| 50 / 2020 (May) | Overall, how satisfied are you with your life nowadays, where 0 means ‘not at all’ and 10 means ‘completely’? | CAWI |
| 50.5 / 2020 (September/October) | Overall, how satisfied are you with your life nowadays, where 0 means ‘not at all’ and 10 means ‘completely’? | CAWI |
| 51 / 2021 (February/March) | Overall, how satisfied are you with your life nowadays, where 0 means ‘not at all’ and 10 means ‘completely’? | CAWI + CATI |

*Note.* CASI: computer-assisted self-interview; CATI: computer-assisted telephone interview; CAWI: computer-assisted web-interview.

## Appendix S2. Distribution of responses to financial situation and time use variables.

The figures below show the distribution of responses to financial situation and time use variables across the COVID-19 survey waves. Due to its reasonably symmetric distribution, financial situation was introduced in the models as a continuous variable in the models. Information on time use was only collected in waves 1 and 2 of the COVID-19 survey, although information on time spent working at the COVID-19 survey wave 3 was approximated using the self-report of hours spent working per week divided by five. A vertical red dashed line represents the values used as thresholds to recode the time use variables into categorical variables.


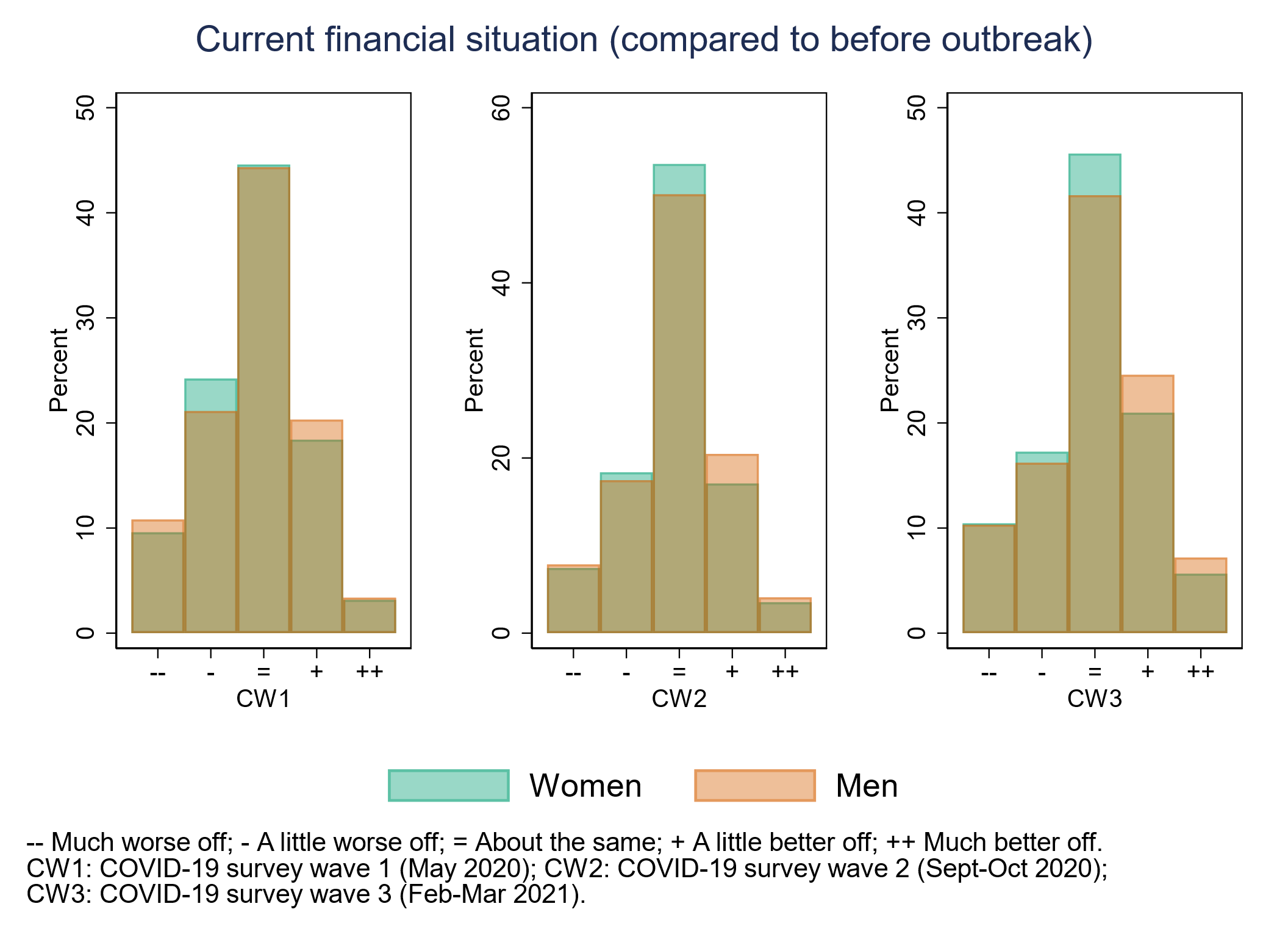


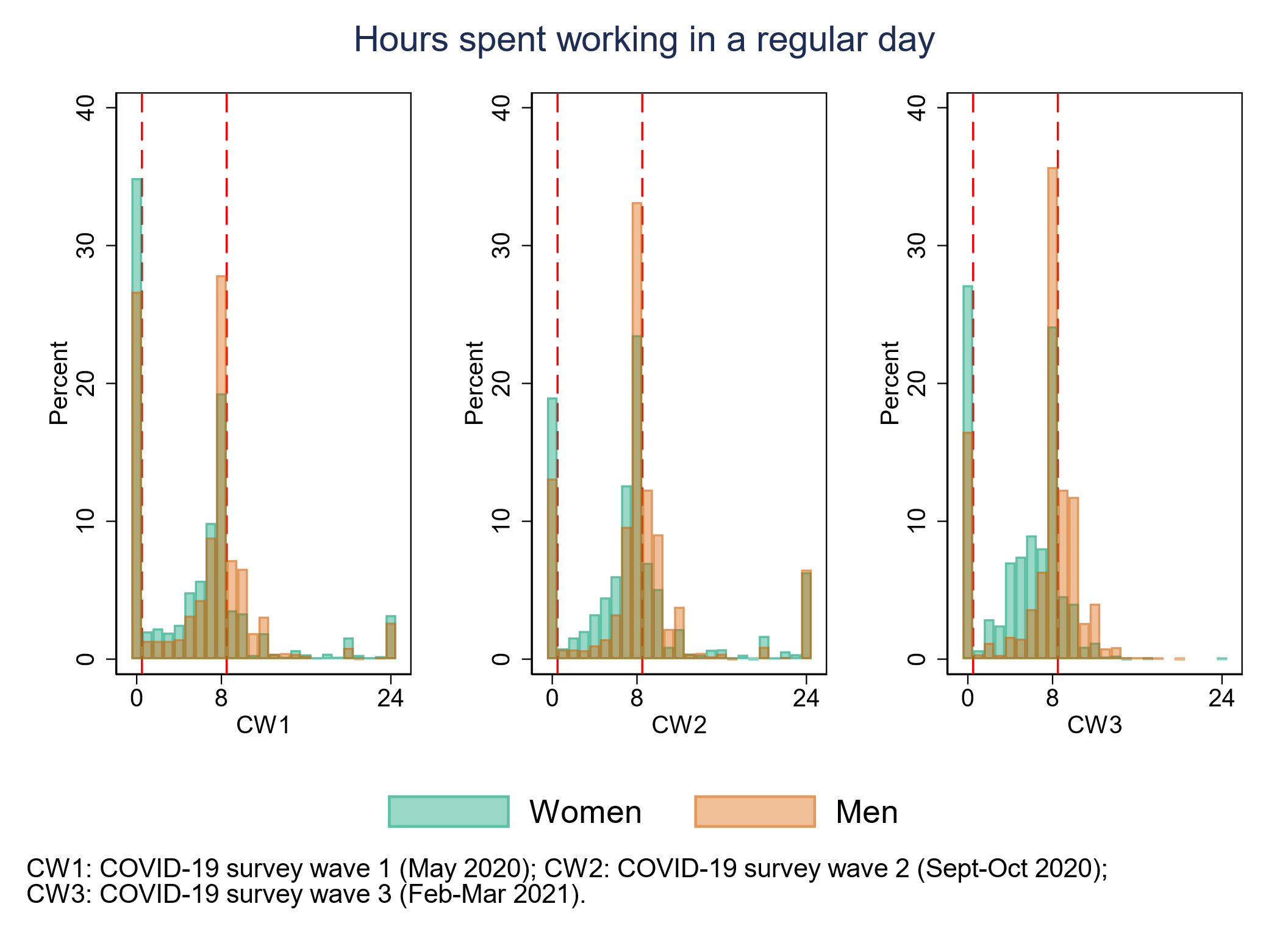


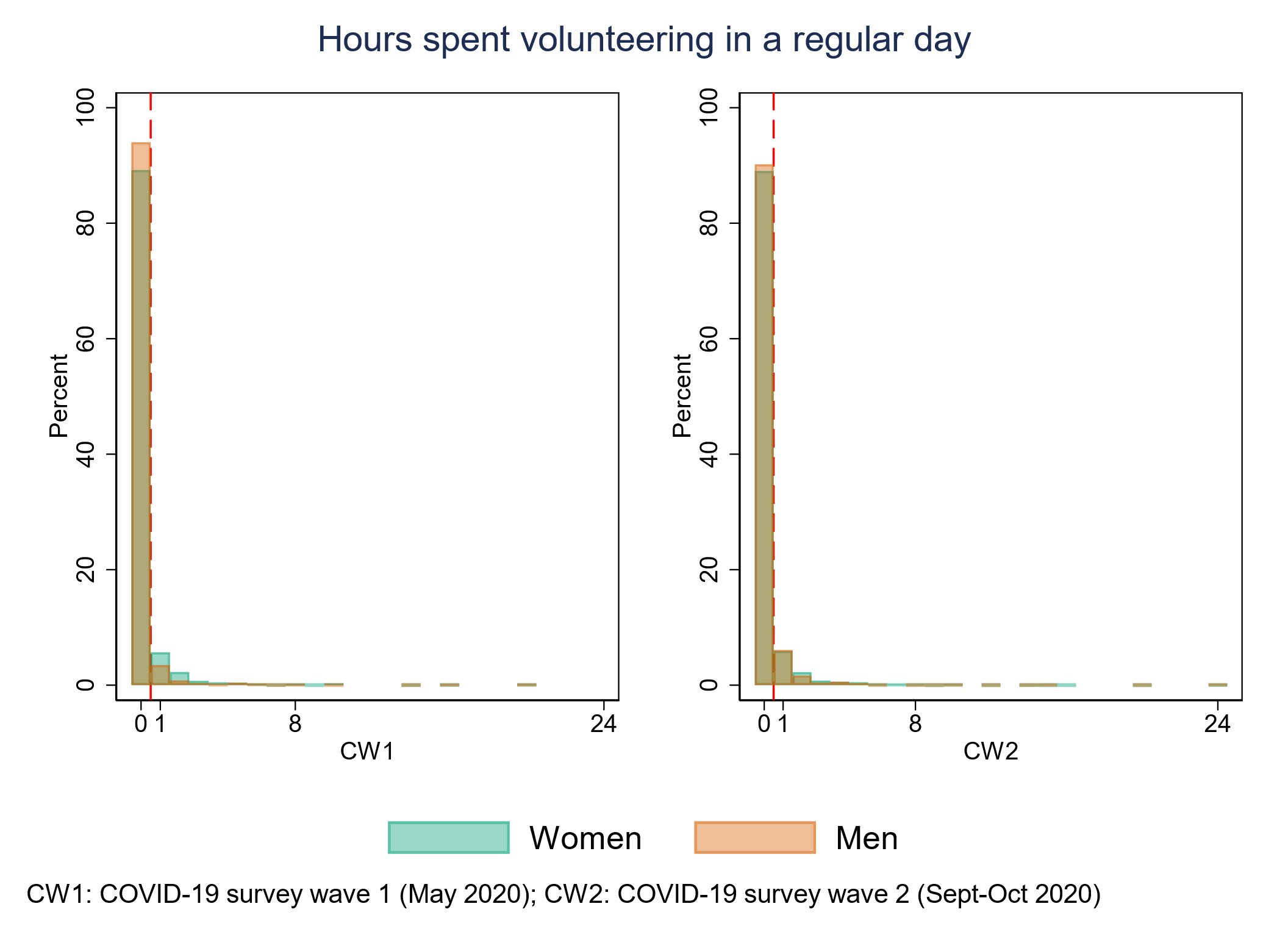


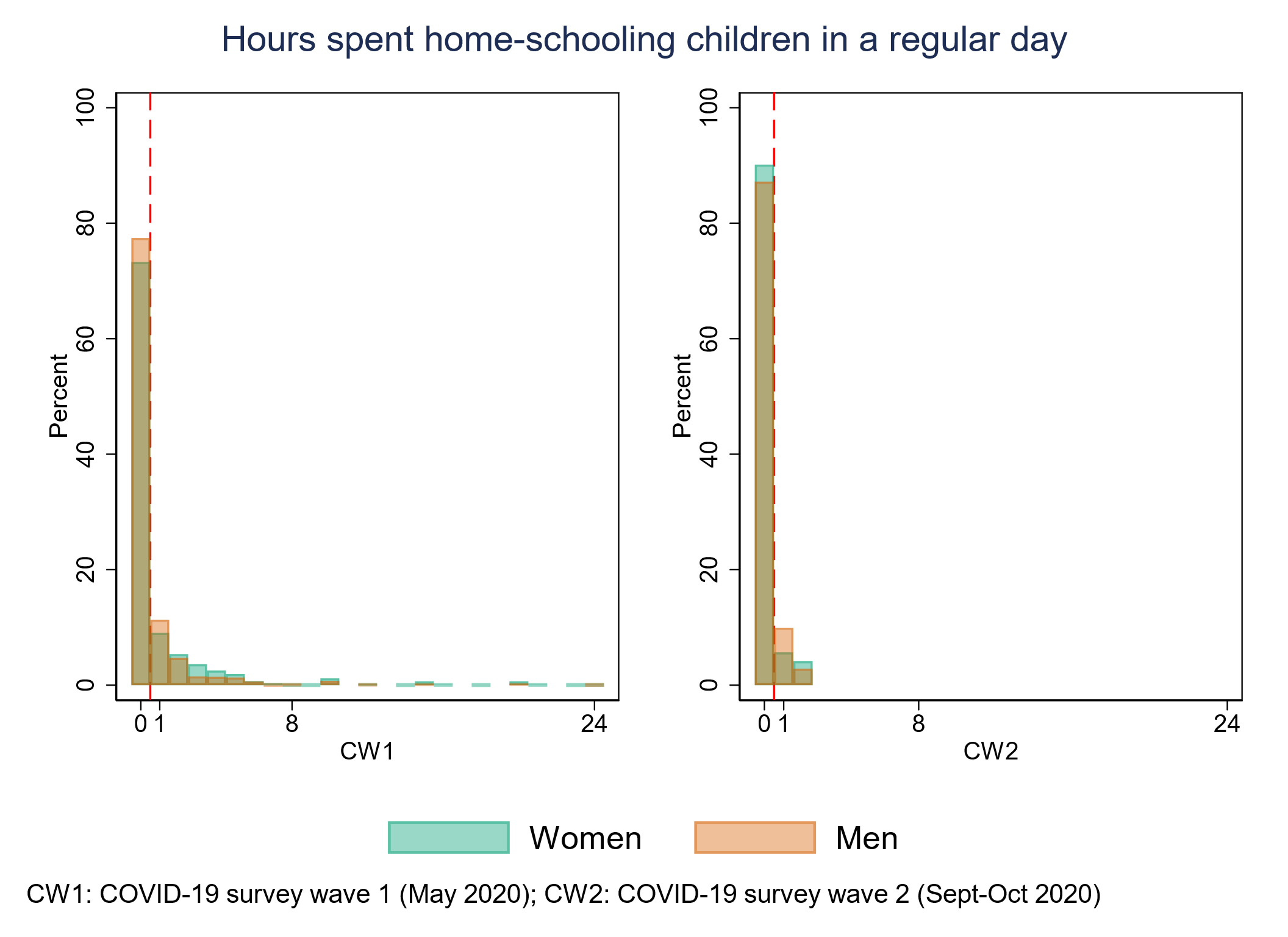


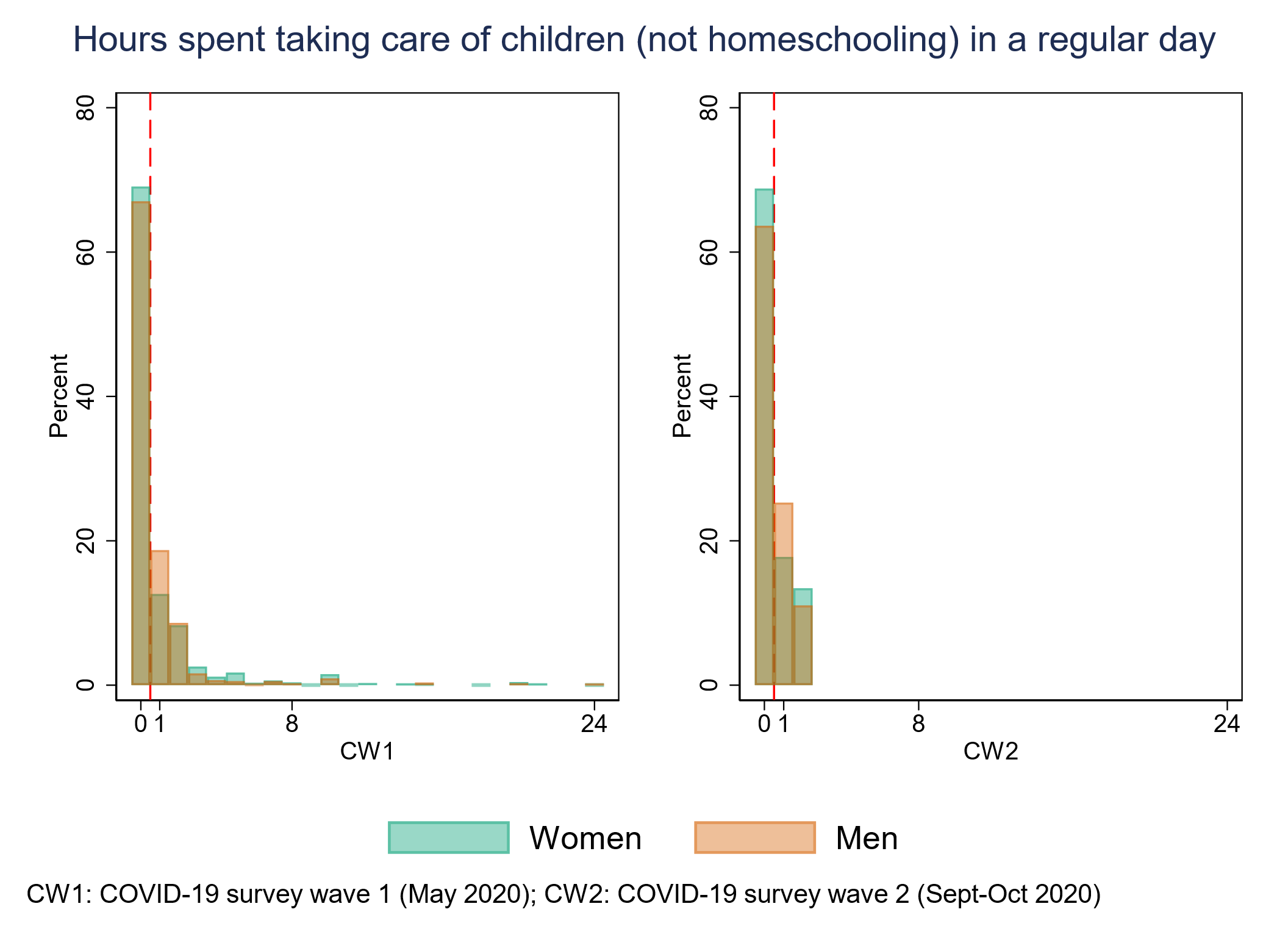


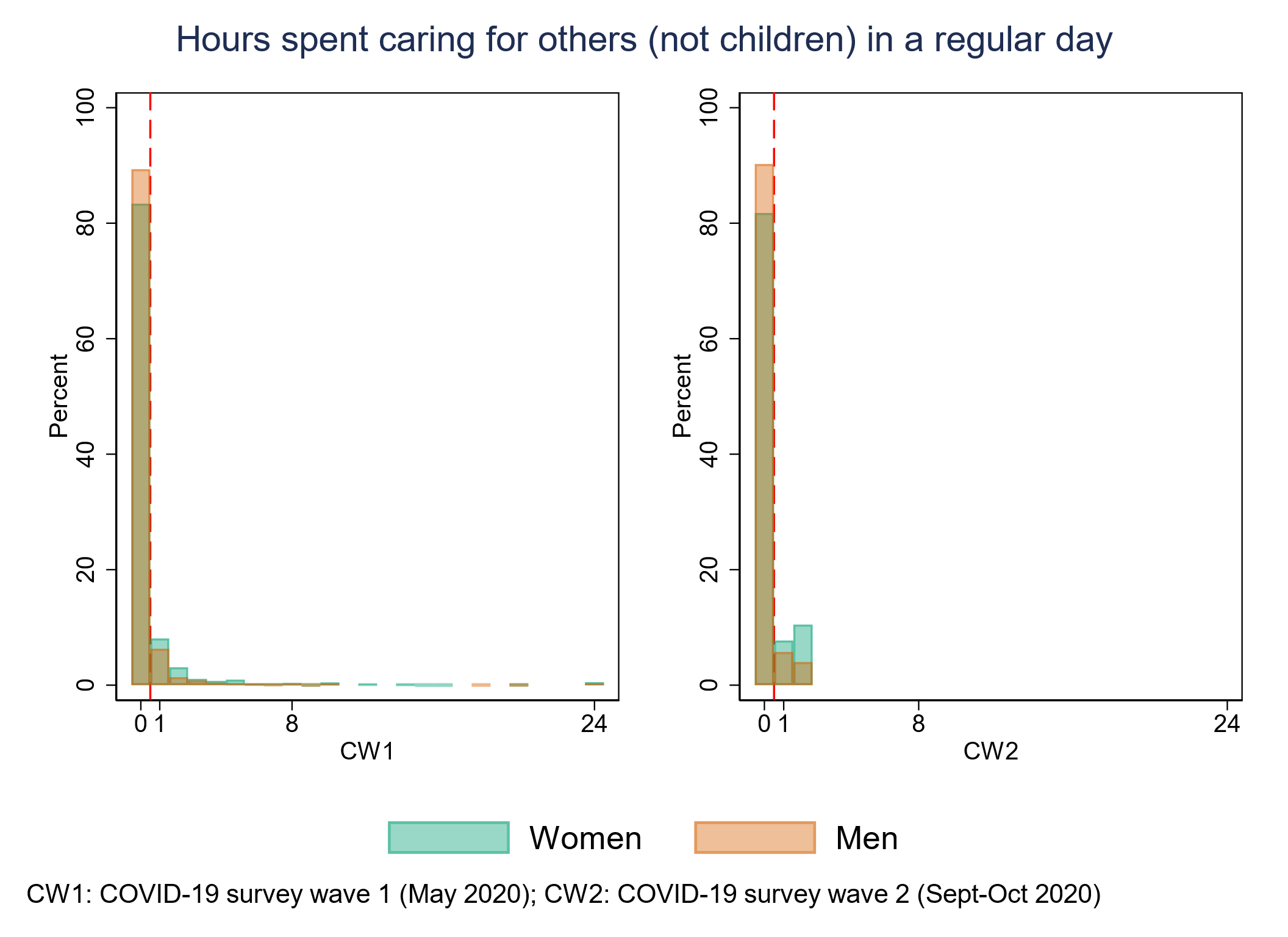


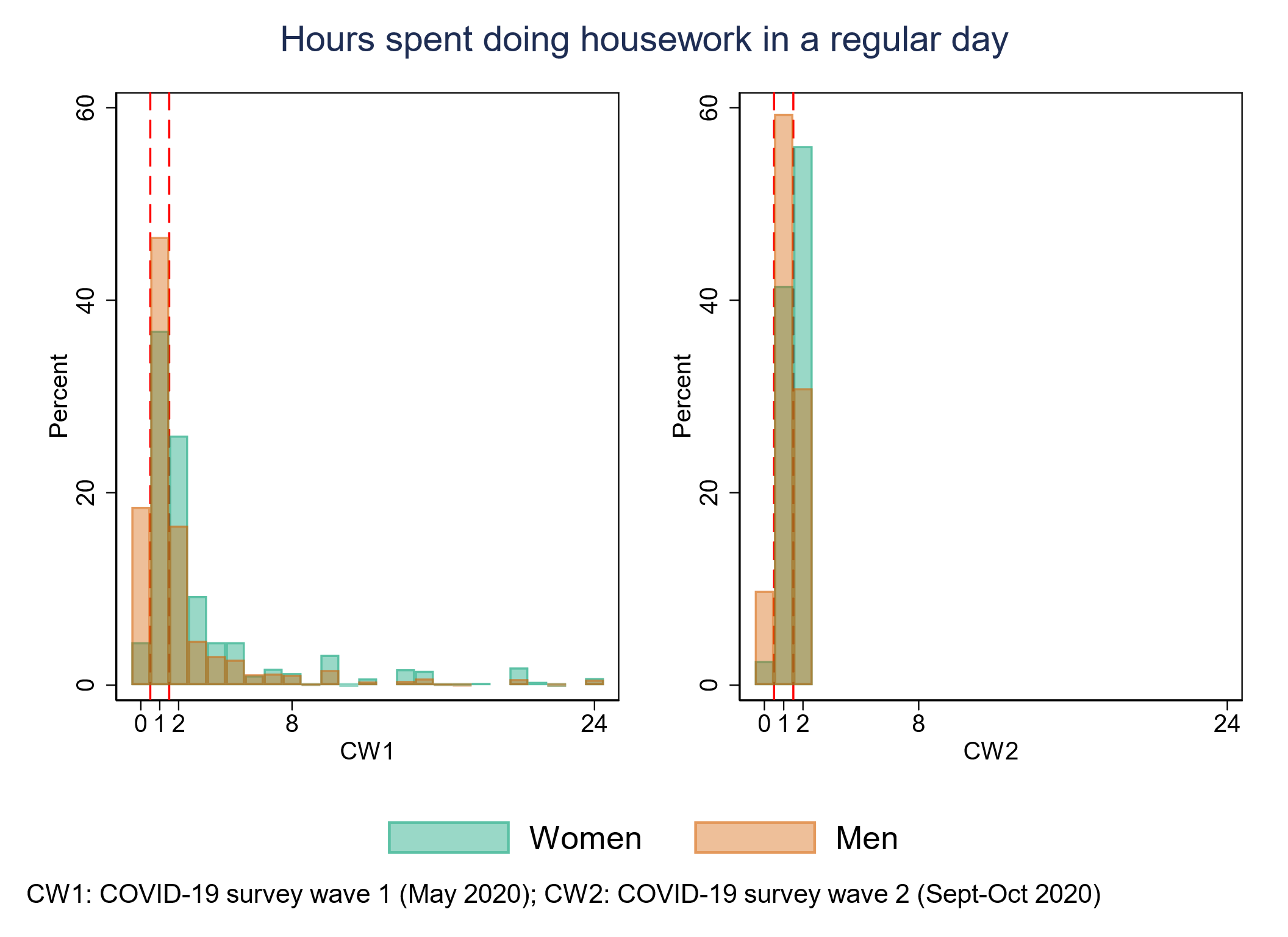


## Appendix S3. Additional details on the rationale for the analytical approach.

Within the Latent Growth Curve Modelling framework, latent variables capturing the change in the trajectories are measured by the repeated observations. The general formalisation of an unconditional LGCM can be summarised into the matrix equation:

$$\mathbf{y}_{i}= \boldsymbol{\Lambda\eta}_{i}+\boldsymbol{\varepsilon}_{i}$$

Where $\mathbf{y}_{i}$ is a vector representing the repeated observations of the outcome of interest for each individual *i*; $\boldsymbol{\Lambda}$ is a matrix capturing the factor loadings for each latent variable (intercept and slopes) at each time point; $\boldsymbol{\eta}_{i}$ is a vector representing the different latent variables for each individual *i*; and $\boldsymbol{\varepsilon}_{i}$ is a vector representing the errors at each time point for each individual *i*.

Due to the multiple-groups approach used, the different terms in the matrix equation above also vary by group (women or men). The model comparison approach documented in **Appendix S7** was aimed at identifying a still tenable yet more parsimonious trajectory model as well as at detecting the growth parameters that were substantially different across groups, the magnitude and significance of which was tested by means of Wald tests of equality of coefficients.

In order to understand to what extent the differences in the growth parameters across women and men during the pandemic (i.e., in the second segment of the trajectories) could be at least partly due to differences in the way in which they used their time during the pandemic, we first included the time-use variables in the latent growth curve models as predictors of the concurrent observations. The updated matrix equation capturing the inclusion of these time-specific variables is:

$$\mathbf{y}_{i}= \boldsymbol{\Lambda\eta}_{i}+\mathbf{KZ}_{i}+\boldsymbol{\varepsilon}_{i}$$

Where $\mathbf{Z}_{i}$ is a matrix containing the time-specific covariates for each individual *i*, and $\mathbf{K}$ is a matrix containing the regression coefficients linking those covariates to the repeated measures.

Any deviance from the general trajectory that could be accounted for by the effect of the included variable(s) would be captured by the time-specific covariate(s) and in turn be reflected in the estimated latent variables, which would capture the trajectory net of the effect of the included variable(s). If the time-use variables were at least partly responsible for the changes in life satisfaction, the resulting trajectory within the group (women or men) would reflect that (e.g., by attenuating the accelerated decline). Then, if the observed between-group differences in life satisfaction were, at least partly, due to the way in which these two groups spent their time, this would be reflected in a smaller difference in the coefficient representing the accelerated decline across the two groups, as tested by the Wald tests of equality of coefficients.

Finally, additional time-specific variables representing key aspects of the financial, occupational, and living arrangements of the individuals (i.e., financial situation, working from home, keyworker status, and living with any dependent child or young person aged ≤16) were included in subsequent models in order to adjust for potential confounders of the relationship between time use and life satisfaction.

The effect of the time-specific variables on life satisfaction was allowed to vary by gender and time. Regarding gender, this more complex yet flexible approach allowed us to avoid making an assumption akin to the “no exposure-mediator interaction” one that has been documented in the literature on causal/formal mediation analysis (for an example, see VanderWeele, T. J. (2016). Mediation analysis: a practitioner's guide. *Annual review of public health*, *37*(1), 17-32.). Regarding time, this was to accommodate the complexities arising from the COVID-19 pandemic, chiefly the presence and absence of lockdowns, as reported in the Methods section, and their impact on the curtailment, availability, or necessity of different activities (i.e., working, volunteering, home-schooling, caring, housework), as well as their potential relationship with life satisfaction.

As an exploratory analysis, gender differences in the relationship between the time spent in different activities and life satisfaction levels at each of the COVID-19 survey waves were tested using Wald tests of equality of coefficients. The null hypothesis of these Wald tests was that the difference between each pair of regression coefficients representing the relationship between each time-use variable and life satisfaction at each time point (e.g., the regression coefficient of working more than 8 hours on life satisfaction at age 50) across women and men was equal to zero.

## Appendix S4. Observed (unweighted) life satisfaction levels across a random (n=200) subset of cohort members over time.


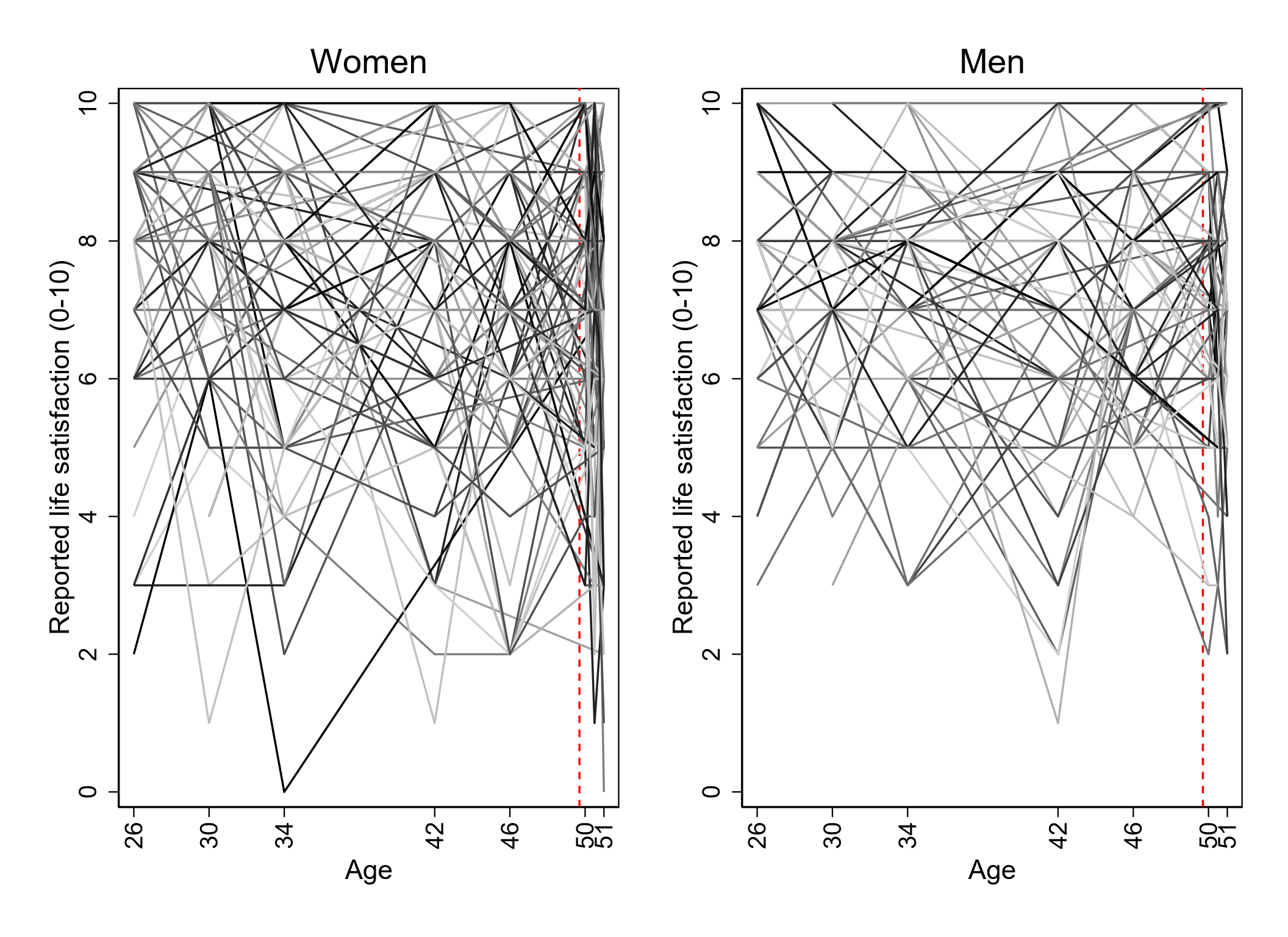


*Note.* The vertical red dashed line represents the pandemic onset.

## Appendix S5. Mean (weighted) observed life satisfaction levels over time.


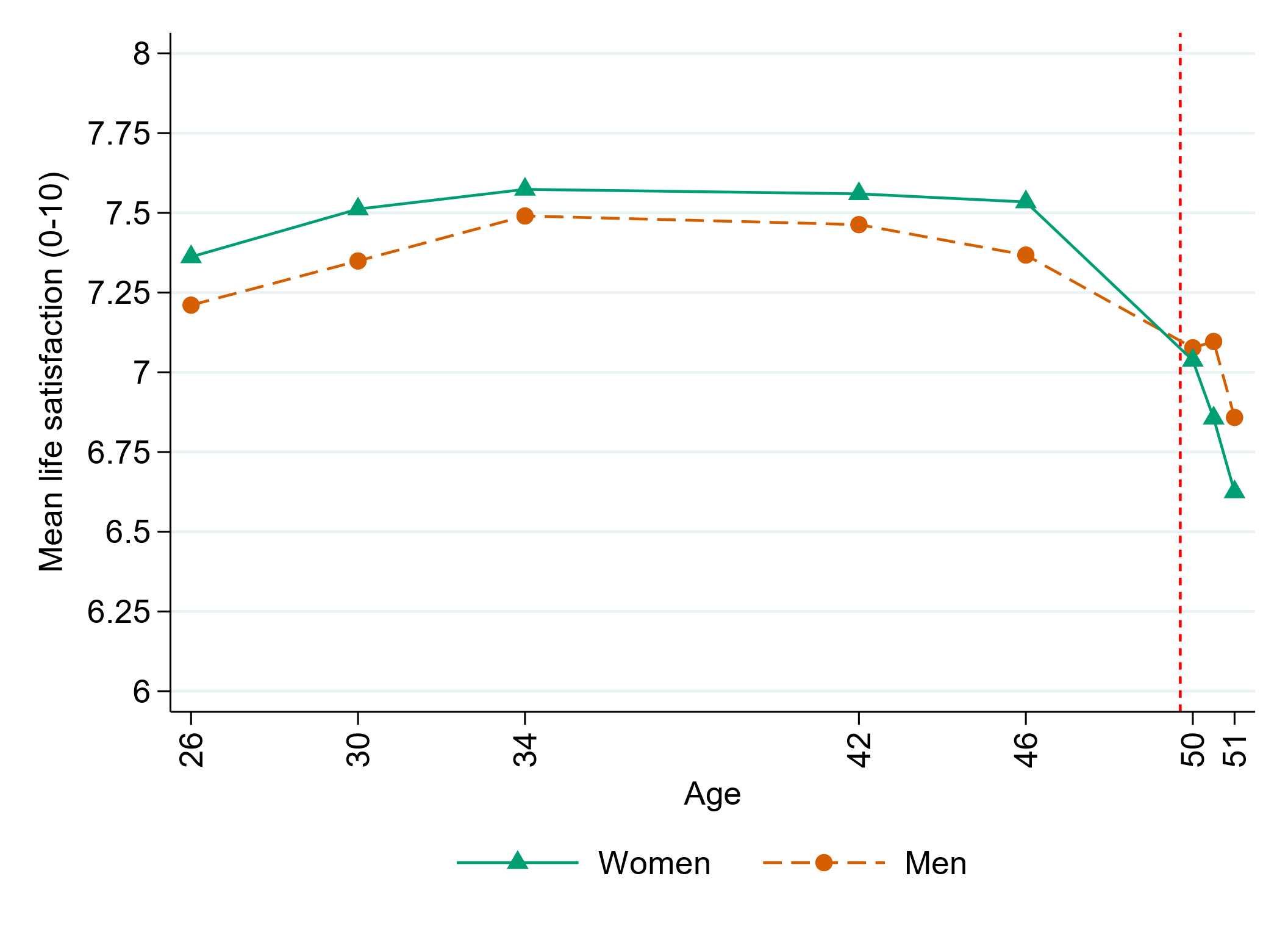


*Note.* The vertical red dashed line represents the pandemic onset.

## Appendix S6. Detailed information on the time-specific covariates (time use, financial situation, working from home, keyworker status, and dependent children or young people in the household) during the COVID-19 pandemic.

|  | **Age 50 (May 2020)** | | | | | |  | **Age 50.5 (Sep/Oct 2020)** | | | | | |  | **Age 51 (Feb/Mar 2021)** | | | | | |
| --- | --- | --- | --- | --- | --- | --- | --- | --- | --- | --- | --- | --- | --- | --- | --- | --- | --- | --- | --- | --- |
|  | **Men  (N=1599)** | | **Women (N=2309)** | | **Overall (N=3908)** | |  | **Men (N=2102)** | | **Women  (N=2895)** | | **Overall  (N=4997)** | |  | **Men  (N=2312)** | | **Women  (N=3132)** | | **Overall  (N=5444)** | |
|  | **N obs.** | **%** | **N obs.** | **%** | **N obs.** | **%** |  | **N obs.** | **%** | **N obs.** | **%** | **N obs.** | **%** |  | **N obs.** | **%** | **N obs.** | **%** | **N obs.** | **%** |
| **Time spent working** |  |  |  |  |  |  |  |  |  |  |  |  |  |  |  |  |  |  |  |  |
| 0 hours | 423 | 26.5 | 798 | 34.6 | 1221 | 31.2 |  | 262 | 12.5 | 530 | 18.3 | 792 | 15.8 |  | 377 | 16.3 | 843 | 26.9 | 1220 | 22.4 |
| 1-8 hours | 786 | 49.2 | 1106 | 47.9 | 1892 | 48.4 |  | 1010 | 48.0 | 1515 | 52.3 | 2525 | 50.5 |  | 1157 | 50.0 | 1914 | 61.1 | 3071 | 56.4 |
| More than 8 hours | 378 | 23.6 | 383 | 16.6 | 761 | 19.5 |  | 728 | 34.6 | 747 | 25.8 | 1475 | 29.5 |  | 752 | 32.5 | 351 | 11.2 | 1103 | 20.3 |
| *Missing* | *12* | *0.8* | *22* | *1.0* | *34* | *0.9* |  | *102* | *4.9* | *103* | *3.6* | *205* | *4.1* |  | *26* | *1.1* | *24* | *0.8* | *50* | *0.9* |
| **Time spent volunteering** |  |  |  |  |  |  |  |  |  |  |  |  |  |  |  |  |  |  |  |  |
| 0 hours | 1492 | 93.3 | 2040 | 88.3 | 3532 | 90.4 |  | 1804 | 85.8 | 2486 | 85.9 | 4290 | 85.9 |  |  |  |  |  |  |  |
| 1+ hours | 95 | 5.9 | 247 | 10.7 | 342 | 8.8 |  | 196 | 9.3 | 306 | 10.6 | 502 | 10.0 |  |  |  |  |  |  |  |
| *Missing* | *12* | *0.8* | *22* | *1.0* | *34* | *0.9* |  | *102* | *4.9* | *103* | *3.6* | *205* | *4.1* |  |  |  |  |  |  |  |
| **Time spent home-schooling** |  |  |  |  |  |  |  |  |  |  |  |  |  |  |  |  |  |  |  |  |
| 0 hours | 1229 | 76.9 | 1676 | 72.6 | 2905 | 74.3 |  | 1744 | 83.0 | 2517 | 86.9 | 4261 | 85.3 |  |  |  |  |  |  |  |
| 1+ hours | 358 | 22.4 | 611 | 26.5 | 969 | 24.8 |  | 256 | 12.2 | 275 | 9.5 | 531 | 10.6 |  |  |  |  |  |  |  |
| *Missing* | *12* | *0.8* | *22* | *1.0* | *34* | *0.9* |  | *102* | *4.9* | *103* | *3.6* | *205* | *4.1* |  |  |  |  |  |  |  |
| **Time spent caring for children** |  |  |  |  |  |  |  |  |  |  |  |  |  |  |  |  |  |  |  |  |
| 0 hours | 1064 | 66.5 | 1580 | 68.4 | 2644 | 67.7 |  | 1273 | 60.6 | 1921 | 66.4 | 3194 | 63.9 |  |  |  |  |  |  |  |
| 1+ hours | 523 | 32.7 | 707 | 30.6 | 1230 | 31.5 |  | 727 | 34.6 | 871 | 30.1 | 1598 | 32.0 |  |  |  |  |  |  |  |
| *Missing* | *12* | *0.8* | *22* | *1.0* | *34* | *0.9* |  | *102* | *4.9* | *103* | *3.6* | *205* | *4.1* |  |  |  |  |  |  |  |
| **Time spent caring for others** |  |  |  |  |  |  |  |  |  |  |  |  |  |  |  |  |  |  |  |  |
| 0 hours | 1418 | 88.7 | 1907 | 82.6 | 3325 | 85.1 |  | 1805 | 85.9 | 2283 | 78.9 | 4088 | 81.8 |  |  |  |  |  |  |  |
| 1+ hours | 169 | 10.6 | 380 | 16.5 | 549 | 14.0 |  | 195 | 9.3 | 509 | 17.6 | 704 | 14.1 |  |  |  |  |  |  |  |
| *Missing* | *12* | *0.8* | *22* | *1.0* | *34* | *0.9* |  | *102* | *4.9* | *103* | *3.6* | *205* | *4.1* |  |  |  |  |  |  |  |
| **Time spent doing housework** |  |  |  |  |  |  |  |  |  |  |  |  |  |  |  |  |  |  |  |  |
| 0 hours | 294 | 18.4 | 102 | 4.4 | 396 | 10.1 |  | 196 | 9.3 | 70 | 2.4 | 266 | 5.3 |  |  |  |  |  |  |  |
| Up to 1 hour | 739 | 46.2 | 842 | 36.5 | 1581 | 40.5 |  | 1135 | 54.0 | 1040 | 35.9 | 2175 | 43.5 |  |  |  |  |  |  |  |
| More than 1 hour | 554 | 34.6 | 1343 | 58.2 | 1897 | 48.5 |  | 669 | 31.8 | 1682 | 58.1 | 2351 | 47.0 |  |  |  |  |  |  |  |
| *Missing* | *12* | *0.8* | *22* | *1.0* | *34* | *0.9* |  | *102* | *4.9* | *103* | *3.6* | *205* | *4.1* |  |  |  |  |  |  |  |
| **Financial situation compared to before the outbreak** |  |  |  |  |  |  |  |  |  |  |  |  |  |  |  |  |  |  |  |  |
| Much worse off | 173 | 10.8 | 221 | 9.6 | 394 | 10.1 |  | 165 | 7.8 | 215 | 7.4 | 380 | 7.6 |  | 238 | 10.3 | 327 | 10.4 | 565 | 10.4 |
| Little worse off | 338 | 21.1 | 558 | 24.2 | 896 | 22.9 |  | 367 | 17.5 | 531 | 18.3 | 898 | 18.0 |  | 374 | 16.2 | 540 | 17.2 | 914 | 16.8 |
| About the same | 709 | 44.3 | 1027 | 44.5 | 1736 | 44.4 |  | 1053 | 50.1 | 1549 | 53.5 | 2602 | 52.1 |  | 961 | 41.6 | 1427 | 45.6 | 2388 | 43.9 |
| Little better off | 325 | 20.3 | 424 | 18.4 | 749 | 19.2 |  | 430 | 20.5 | 494 | 17.1 | 924 | 18.5 |  | 567 | 24.5 | 656 | 20.9 | 1223 | 22.5 |
| Much better off | 54 | 3.4 | 73 | 3.2 | 127 | 3.2 |  | 86 | 4.1 | 102 | 3.5 | 188 | 3.8 |  | 166 | 7.2 | 177 | 5.7 | 343 | 6.3 |
| *Missing* | *0* | *0.0* | *6* | *0.3* | *6* | *0.2* |  | *1* | *0.0* | *4* | *0.1* | *5* | *0.1* |  | *6* | *0.3* | *5* | *0.2* | *11* | *0.2* |
| **Works from home** |  |  |  |  |  |  |  |  |  |  |  |  |  |  |  |  |  |  |  |  |
| Never | 935 | 58.5 | 1406 | 60.9 | 2341 | 59.9 |  | 1209 | 57.5 | 1863 | 64.4 | 3072 | 61.5 |  | 1279 | 55.3 | 1888 | 60.3 | 3167 | 58.2 |
| Part or all of the time | 663 | 41.5 | 885 | 38.3 | 1548 | 39.6 |  | 890 | 42.3 | 1029 | 35.5 | 1919 | 38.4 |  | 1028 | 44.5 | 1243 | 39.7 | 2271 | 41.7 |
| *Missing* | *1* | *0.1* | *18* | *0.8* | *19* | *0.5* |  | *3* | *0.1* | *3* | *0.1* | *6* | *0.1* |  | *5* | *0.2* | *1* | *0.0* | *6* | *0.1* |
| **Keyworker** |  |  |  |  |  |  |  |  |  |  |  |  |  |  |  |  |  |  |  |  |
| No | 1071 | 67.0 | 1448 | 62.7 | 2519 | 64.5 |  | 1352 | 64.3 | 1678 | 58.0 | 3030 | 60.6 |  | 1424 | 61.6 | 1684 | 53.8 | 3108 | 57.1 |
| Yes | 525 | 32.8 | 843 | 36.5 | 1368 | 35.0 |  | 746 | 35.5 | 1212 | 41.9 | 1958 | 39.2 |  | 881 | 38.1 | 1444 | 46.1 | 2325 | 42.7 |
| *Missing* | *3* | *0.2* | *18* | *0.8* | *21* | *0.5* |  | *4* | *0.2* | *5* | *0.2* | *9* | *0.2* |  | *7* | *0.3* | *4* | *0.1* | *11* | *0.2* |
| **Dependent children or young people in the household** |  |  |  |  |  |  |  |  |  |  |  |  |  |  |  |  |  |  |  |  |
| No | 913 | 57.1 | 1381 | 59.8 | 2294 | 58.7 |  | 1271 | 60.5 | 1855 | 64.1 | 3126 | 62.6 |  | 1537 | 66.5 | 2158 | 68.9 | 3695 | 67.9 |
| Yes | 685 | 42.8 | 926 | 40.1 | 1611 | 41.2 |  | 826 | 39.3 | 1027 | 35.5 | 1853 | 37.1 |  | 761 | 32.9 | 959 | 30.6 | 1720 | 31.6 |
| *Missing* | *1* | *0.1* | *2* | *0.1* | *3* | *0.1* |  | *5* | *0.2* | *13* | *0.4* | *18* | *0.4* |  | *14* | *0.6* | *15* | *0.5* | *29* | *0.5* |

*Note.* Unweighted results. N obs: total number of observations.

## Appendix S7. Fit indices for the latent growth curve models estimated to identify the optimal functional form in the overall, women, and men samples.

| **Women** |  |  |  |  |  |  |  |  |  |  |  |  |  |  |  |
| --- | --- | --- | --- | --- | --- | --- | --- | --- | --- | --- | --- | --- | --- | --- | --- |
| **Model** | **Comp. model for Δχ^2^** | **χ^2^** | **df** | **Scaling correction factor** | **AIC** | **BIC** | **RMSEA** | **RMSEA LB** | **RMSEA UB** | **CFI** | **TLI** | **SRMR** | **Δχ^2^** | **df** | **Δχ^2^ *p*-value** |
| a) No change / Intercepts-only |  | 669.1 | 34 | 2.13 | 80093 | 80156 | 0.070 | 0.066 | 0.075 | 0.666 | 0.725 | 0.146 |  |  |  |
| b) Linear change | a | 342.1 | 31 | 2.09 | 79390 | 79471 | 0.051 | 0.047 | 0.056 | 0.837 | 0.852 | 0.071 | 279.24 | 3 | <0.001 |
| c) Quadratic change | b | 122.5 | 27 | 2.12 | 78943 | 79049 | 0.031 | 0.025 | 0.036 | 0.95 | 0.948 | 0.050 | 241.21 | 4 | <0.001 |
| d) Cubic change | c | 88.9 | 22 | 2.06 | 78876 | 79013 | 0.028 | 0.022 | 0.035 | 0.965 | 0.955 | 0.044 | 32.12 | 5 | <0.001 |
| e) Piecewise: linear + linear | a | 72.3 | 27 | 2.10 | 78835 | 78941 | 0.021 | 0.015 | 0.027 | 0.976 | 0.975 | 0.038 | 567.01 | 7 | <0.001 |
| f) Piecewise: quadratic + linear | e | 61.2 | 22 | 1.98 | 78814 | 78952 | 0.022 | 0.015 | 0.028 | 0.979 | 0.974 | 0.037 | 11.66 | 5 | 0.040 |
| **g) Piecewise: quadratic + quadratic** | **f** | **42.6** | **16** | **1.83** | **78783** | **78958** | **0.021** | **0.013** | **0.029** | **0.986** | **0.976** | **0.032** | **18.16** | **6** | **0.005** |
| h) Free / latent basis | a | 145.5 | 25 | 2.08 | 78990 | 79108 | 0.036 | 0.030 | 0.041 | 0.937 | 0.929 | 0.048 | 494.75 | 9 | <0.001 |
|  |  |  |  |  |  |  |  |  |  |  |  |  |  |  |  |
| **Men** |  |  |  |  |  |  |  |  |  |  |  |  |  |  |  |
| **Model** | **Comp. model for Δχ^2^** | **χ^2^** | **df** | **Scaling correction factor** | **AIC** | **BIC** | **RMSEA** | **RMSEA LB** | **RMSEA UB** | **CFI** | **TLI** | **SRMR** | **Δχ^2^** | **df** | **Δχ^2^ *p*-value** |
| a) No change / Intercepts-only |  | 350.7 | 34 | 2.20 | 55709 | 55769 | 0.056 | 0.051 | 0.061 | 0.813 | 0.846 | 0.124 |  |  |  |
| b) Linear change | a | 174.4 | 31 | 2.11 | 55310 | 55388 | 0.039 | 0.034 | 0.045 | 0.916 | 0.924 | 0.063 | 128.93 | 3 | <0.001 |
| c) Quadratic change | b | 56.1 | 27 | 2.01 | 55064 | 55166 | 0.019 | 0.012 | 0.026 | 0.983 | 0.982 | 0.032 | 91.64 | 4 | <0.001 |
| d) Cubic change | c | 27.9 | 22 | 1.86 | 55013 | 55145 | 0.010 | <0.001 | 0.019 | 0.997 | 0.996 | 0.019 | 22.80 | 5 | <0.001 |
| e) Piecewise: linear + linear | a | 59.9 | 27 | 2.00 | 55071 | 55173 | 0.020 | 0.013 | 0.027 | 0.981 | 0.980 | 0.034 | 219.34 | 7 | <0.001 |
| f) Piecewise: quadratic + linear | e | 34.1 | 22 | 1.81 | 55023 | 55155 | 0.014 | 0.001 | 0.022 | 0.993 | 0.991 | 0.023 | 20.48 | 5 | 0.001 |
| **g) Piecewise: quadratic + quadratic** | **f** | **18.8** | **16** | **1.94** | **55009** | **55177** | **0.008** | **<0.001** | **0.019** | **0.998** | **0.997** | **0.021** | **17.25** | **6** | **0.008** |
| h) Free / latent basis | a | 105.1 | 25 | 2.00 | 55165 | 55279 | 0.033 | 0.027 | 0.039 | 0.953 | 0.947 | 0.039 | 203.71 | 9 | <0.001 |
|  |  |  |  |  |  |  |  |  |  |  |  |  |  |  |  |
| **Overall** |  |  |  |  |  |  |  |  |  |  |  |  |  |  |  |
| **Model** | **Comp. model for Δχ^2^** | **χ^2^** | **df** | **Scaling correction factor** | **AIC** | **BIC** | **RMSEA** | **RMSEA LB** | **RMSEA UB** | **CFI** | **TLI** | **SRMR** | **Δχ^2^** | **df** | **Δχ^2^ *p*-value** |
| a) No change / Intercepts-only |  | 837.2 | 34 | 2.33 | 134545 | 134613 | 0.059 | 0.056 | 0.063 | 0.758 | 0.801 | 0.130 |  |  |  |
| b) Linear change | a | 416.3 | 31 | 2.23 | 133528 | 133616 | 0.043 | 0.039 | 0.047 | 0.884 | 0.895 | 0.059 | 303.96 | 3 | <0.001 |
| c) Quadratic change | b | 118.5 | 27 | 2.23 | 132870 | 132986 | 0.022 | 0.018 | 0.027 | 0.972 | 0.971 | 0.033 | 297.80 | 4 | <0.001 |
| d) Cubic change | c | 64.6 | 22 | 2.16 | 132756 | 132906 | 0.017 | 0.012 | 0.022 | 0.987 | 0.984 | 0.025 | 49.14 | 5 | <0.001 |
| e) Piecewise: linear + linear | a | 81.8 | 27 | 2.22 | 132788 | 132904 | 0.017 | 0.013 | 0.022 | 0.984 | 0.983 | 0.026 | 642.30 | 7 | <0.001 |
| f) Piecewise: quadratic + linear | e | 47.4 | 22 | 2.10 | 132716 | 132866 | 0.013 | 0.008 | 0.018 | 0.992 | 0.99 | 0.021 | 29.86 | 5 | <0.001 |
| **g) Piecewise: quadratic + quadratic** | **f** | **19.5** | **16** | **2.07** | **132669** | **132860** | **0.006** | **<0.001** | **0.013** | **0.999** | **0.998** | **0.014** | **27.14** | **6** | **<0.001** |
| h) Free / latent basis | a | 201.2 | 25 | 2.23 | 133060 | 133189 | 0.032 | 0.028 | 0.036 | 0.947 | 0.941 | 0.039 | 575.97 | 9 | <0.001 |

*Note*. Models estimated using non-response weights and full information maximum likelihood under MLR estimation. Favoured models are highlighted in boldface. AIC: Akaike Information Criterion; BIC: Bayesian Information Criterion; CFI: Comparative Fit Index; Comp. model for Δχ^2^: comparison model used in the Satorra-Bentler scaled robust chi-square difference test; df: degrees of freedom; Δχ^2^: chi-square difference test statistic; RMSEA: root mean square error of approximation (LB: lower bound; UB: upper bound); SRMR: standardised root mean square residual; TLI: Tucker-Lewis Index; χ^2^: chi-square statistic.

## Appendix S8. Fit indices for the multiple groups latent growth curve models estimated to identify the most parsimonious growth model across groups.

| **Model** | **Constraints** | **Comp. model for Δχ^2^** | **χ^2^** | **df** | **Scaling correction factor** | **AIC** | **BIC** | **RMSEA** | **RMSEA LB** | **RMSEA UP** | **CFI** | **TLI** | **SRMR** | **Δχ^2^** | **df** | **Δχ^2^ *p*-value** |
| --- | --- | --- | --- | --- | --- | --- | --- | --- | --- | --- | --- | --- | --- | --- | --- | --- |
| a | Baseline / no constraints |  | 60.7 | 32 | 1.88 | 133793 | 134175 | 0.016 | 0.010 | 0.022 | 0.992 | 0.986 | 0.028 |  |  |  |
| b | Equal quad1 mean across groups | a | 61.1 | 33 | 1.90 | 133793 | 134168 | 0.016 | 0.009 | 0.022 | 0.992 | 0.987 | 0.028 | 0.78 | 1 | 0.377 |
| c | b + equal lin1 mean across groups | b | 61.4 | 34 | 1.92 | 133793 | 134161 | 0.015 | 0.009 | 0.022 | 0.992 | 0.987 | 0.028 | 0.70 | 1 | 0.403 |
| d | c + equal lin2 mean across groups | c | 61.8 | 35 | 1.91 | 133791 | 134152 | 0.015 | 0.009 | 0.021 | 0.993 | 0.988 | 0.028 | 0.10 | 1 | 0.752 |
| e | d + equal quad2 mean across groups | d | - |  |  |  |  |  |  |  |  |  |  |  |  |  |
| f | d + quad2 variance fixed to zero in both groups | d | 68.1 | 45 | 1.91 | 133782 | 134076 | 0.012 | 0.006 | 0.018 | 0.994 | 0.992 | 0.029 | 6.30 | 10 | 0.790 |
| g | f + quad1 variance fixed to zero in both groups | f | 81.3 | 53 | 2.04 | 133803 | 134041 | 0.013 | 0.007 | 0.018 | 0.992 | 0.992 | 0.031 | 12.91 | 8 | 0.115 |
| h | g + lin1 variance fixed to zero in both groups | g | 310.3 | 59 | 2.23 | 134316 | 134514 | 0.035 | 0.032 | 0.039 | 0.93 | 0.934 | 0.079 | 134.61 | 6 | <0.001 |
| i | f + equal lin1 variance across groups | g | 80.1 | 54 | 2.07 | 133801 | 134032 | 0.012 | 0.006 | 0.017 | 0.993 | 0.993 | 0.031 | -0.01 | 1 | 1 |
| j | i + lin2 variance fixed to zero in both groups | i | 375.7 | 60 | 2.02 | 134383 | 134574 | 0.039 | 0.036 | 0.043 | 0.913 | 0.918 | 0.069 | 377.78 | 6 | <0.001 |
| k | i + equal lin2 variance across groups | i | 78.8 | 55 | 2.11 | 133799 | 134024 | 0.011 | 0.005 | 0.017 | 0.993 | 0.993 | 0.031 | 0.11 | 1 | 0.740 |
| **l** | **k + equal int variance across groups** | **k** | **80.9** | **56** | **2.13** | **133803** | **134021** | **0.011** | **0.005** | **0.017** | **0.993** | **0.993** | **0.036** | **1.87** | **1** | **0.172** |

*Note*. Models estimated using non-response weights and full information maximum likelihood under MLR estimation. Favoured model is highlighted in boldface. AIC: Akaike Information Criterion; BIC: Bayesian Information Criterion; CFI: Comparative Fit Index; Comp. model for Δχ^2^: comparison model used in the Satorra-Bentler scaled robust chi-square difference test; df: degrees of freedom; Δχ^2^: chi-square difference test statistic; int: intercept term; lin1: linear growth term for the first segment of the piecewise model; lin2: linear growth term for the second segment of the piecewise model; quad1: quadratic growth term for the first segment of the piecewise model; quad2: quadratic growth term for the second segment of the piecewise model; RMSEA: root mean square error of approximation (LB: lower bound; UB: upper bound); SRMR: standardised root mean square residual; TLI: Tucker-Lewis Index; χ^2^: chi-square statistic.

## Appendix S9. Results from the Wald tests analysing the difference in the relationship between time-use variables and life satisfaction across women and men, based on fully adjusted multiple group latent growth curve models (n=6,766).

|  | **Point estimate** | **95% CI, LB** | **95% CI, UB** | **p-value** |
| --- | --- | --- | --- | --- |
| **Difference in effect on life satisfaction at age 50** |  |  |  |  |
| Working 1-8 hours | 0.043 | -0.336 | 0.421 | 0.824 |
| Working more than 8 hours | 0.278 | -0.157 | 0.714 | 0.211 |
| Volunteering 1 or more hours | -0.241 | -0.654 | 0.173 | 0.254 |
| Home-schooling 1 or more hours | 0.315 | -0.060 | 0.690 | 0.099 |
| Caring for children 1 or more hours | 0.052 | -0.286 | 0.390 | 0.763 |
| Caring for others 1 or more hours | -0.001 | -0.374 | 0.371 | 0.994 |
| Doing housework 1 hour | -0.283 | -0.674 | 0.107 | 0.155 |
| Doing housework 2 or more hours | -0.209 | -0.636 | 0.217 | 0.336 |
| **Difference in effect on life satisfaction at age 50.5** |  |  |  |  |
| Working 1-8 hours | 0.535 | 0.156 | 0.914 | 0.006 |
| Working more than 8 hours | 0.560 | 0.143 | 0.978 | 0.009 |
| Volunteering 1 or more hours | -0.132 | -0.463 | 0.198 | 0.433 |
| Home-schooling 1 or more hours | -0.046 | -0.495 | 0.403 | 0.841 |
| Caring for children 1 or more hours | 0.020 | -0.293 | 0.334 | 0.898 |
| Caring for others 1 or more hours | -0.486 | -0.884 | -0.087 | 0.017 |
| Doing housework 1 hour | -0.555 | -0.956 | -0.155 | 0.007 |
| Doing housework 2 or more hours | -0.409 | -0.855 | 0.037 | 0.072 |
| **Difference in effect on life satisfaction at age 51** |  |  |  |  |
| Working 1-8 hours | 0.104 | -0.366 | 0.574 | 0.664 |
| Working more than 8 hours | -0.019 | -0.535 | 0.497 | 0.943 |

*Note.* Results based on models estimated using non-response weights and full information maximum likelihood under MLR estimation and adjusted for financial situation, working-from-home and keyworker status, and presence in the household of dependent children or young people aged up to 16 years old. CI: confidence interval; LB: lower bound; UB: upper bound. Effects of time use variables are estimated with no time spent in that activity as a reference category. Wald tests estimate the statistical significance of the null hypothesis of estimate_women_ - estimate_men_ = 0. Therefore, a positive point estimate indicates that the variable has a more positive or less negative effect on women’s life satisfaction, whereas a negative point estimate indicates that the variable has a more positive or less negative effect on men’s life satisfaction.

## Appendix S10. Results from the multiple group latent growth curve models adjusted for interview mode (n=6,766).

|  | **Adjusted for interview mode only** | | | | | | |  | **Fully adjusted *** | | | | | | |
| --- | --- | --- | --- | --- | --- | --- | --- | --- | --- | --- | --- | --- | --- | --- | --- |
|  | **Women** | | |  | **Men** | | |  | **Women** | | |  | **Men** | | |
|  | **Point estimate** | **95% CI, LB** | **95% CI, UB** |  | **Point estimate** | **95% CI, LB** | **95% CI, UB** |  | **Point estimate** | **95% CI, LB** | **95% CI, UB** |  | **Point estimate** | **95% CI, LB** | **95% CI, UB** |
| **Means** |  |  |  |  |  |  |  |  |  |  |  |  |  |  |  |
| Intercept | 7.195 | 7.103 | 7.288 |  | 6.978 | 6.863 | 7.093 |  | 7.188 | 7.093 | 7.283 |  | 6.966 | 6.848 | 7.085 |
| Linear change, first segment | *0.041* | *0.026* | *0.057* |  | *0.041* | *0.026* | *0.057* |  | *0.041* | *0.026* | *0.057* |  | *0.041* | *0.026* | *0.057* |
| Quadratic change, first segment | *-0.002* | *-0.002* | *-0.001* |  | *-0.002* | *-0.002* | *-0.001* |  | *-0.002* | *-0.002* | *-0.001* |  | *-0.002* | *-0.002* | *-0.001* |
| Linear change, second segment | *0.248* | *0.156* | *0.341* |  | *0.248* | *0.156* | *0.341* |  | *0.313* | *-0.051* | *0.677* |  | *0.313* | *-0.051* | *0.677* |
| Quadratic change, second segment | -0.082 | -0.102 | -0.063 |  | -0.061 | -0.081 | -0.041 |  | -0.106 | -0.180 | -0.032 |  | -0.079 | -0.160 | 0.002 |
| **Variances** |  |  |  |  |  |  |  |  |  |  |  |  |  |  |  |
| Intercept | *1.698* | *1.465* | *1.932* |  | *1.698* | *1.465* | *1.932* |  | *1.720* | *1.481* | *1.960* |  | *1.720* | *1.481* | *1.960* |
| Linear change, first segment | *0.116* | *0.086* | *0.147* |  | *0.116* | *0.086* | *0.147* |  | *0.116* | *0.086* | *0.146* |  | *0.116* | *0.086* | *0.146* |
| Quadratic change, first segment | *0* |  |  |  | *0* |  |  |  | *0* |  |  |  | *0* |  |  |
| Linear change, second segment | *1.859* | *1.480* | *2.238* |  | *1.859* | *1.480* | *2.238* |  | *1.801* | *1.427* | *2.175* |  | *1.801* | *1.427* | *2.175* |
| Quadratic change, second segment | *0* |  |  |  | *0* |  |  |  | *0* |  |  |  | *0* |  |  |
| **Standardised covariances (correlations)** |  |  |  |  |  |  |  |  |  |  |  |  |  |  |  |
| Intercept x linear change, first segment | -0.346 | -0.473 | -0.219 |  | -0.326 | -0.456 | -0.197 |  | -0.347 | -0.475 | -0.219 |  | -0.323 | -0.453 | -0.193 |
| Intercept x linear change, second segment | -0.093 | -0.211 | 0.026 |  | -0.103 | -0.267 | 0.060 |  | -0.123 | -0.241 | -0.004 |  | -0.147 | -0.313 | 0.019 |
| Linear change first x second segment | -0.117 | -0.288 | 0.053 |  | -0.132 | -0.326 | 0.062 |  | -0.128 | -0.299 | 0.043 |  | -0.118 | -0.312 | 0.077 |
| **Standardised residual variances** |  |  |  |  |  |  |  |  |  |  |  |  |  |  |  |
| Age 26 | 0.556 | 0.499 | 0.613 |  | 0.550 | 0.489 | 0.611 |  | 0.554 | 0.498 | 0.610 |  | 0.547 | 0.488 | 0.605 |
| Age 30 | 0.564 | 0.518 | 0.610 |  | 0.517 | 0.457 | 0.577 |  | 0.561 | 0.515 | 0.607 |  | 0.512 | 0.452 | 0.572 |
| Age 34 | 0.547 | 0.501 | 0.593 |  | 0.483 | 0.422 | 0.545 |  | 0.542 | 0.496 | 0.588 |  | 0.479 | 0.419 | 0.539 |
| Age 42 | 0.544 | 0.483 | 0.604 |  | 0.507 | 0.452 | 0.562 |  | 0.542 | 0.482 | 0.603 |  | 0.500 | 0.446 | 0.555 |
| Age 46 | 0.390 | 0.316 | 0.464 |  | 0.382 | 0.300 | 0.464 |  | 0.391 | 0.318 | 0.464 |  | 0.381 | 0.305 | 0.457 |
| Age 50 | 0.411 | 0.337 | 0.484 |  | 0.272 | 0.222 | 0.323 |  | 0.409 | 0.348 | 0.470 |  | 0.270 | 0.222 | 0.318 |
| Age 50.5 | 0.298 | 0.256 | 0.340 |  | 0.285 | 0.223 | 0.348 |  | 0.281 | 0.238 | 0.324 |  | 0.289 | 0.231 | 0.347 |
| Age 51 | 0.332 | 0.282 | 0.382 |  | 0.273 | 0.221 | 0.325 |  | 0.322 | 0.277 | 0.367 |  | 0.254 | 0.206 | 0.301 |
| **Effects on life satisfaction at age 50** |  |  |  |  |  |  |  |  |  |  |  |  |  |  |  |
| Working 1-8 hours |  |  |  |  |  |  |  |  | 0.161 | -0.132 | 0.453 |  | 0.118 | -0.129 | 0.365 |
| Working more than 8 hours |  |  |  |  |  |  |  |  | 0.183 | -0.169 | 0.536 |  | -0.088 | -0.353 | 0.177 |
| Volunteering 1 or more hours |  |  |  |  |  |  |  |  | -0.169 | -0.419 | 0.080 |  | 0.090 | -0.242 | 0.421 |
| Home-schooling 1 or more hours |  |  |  |  |  |  |  |  | 0.241 | -0.060 | 0.541 |  | -0.070 | -0.299 | 0.159 |
| Caring for children 1 or more hours |  |  |  |  |  |  |  |  | -0.069 | -0.335 | 0.197 |  | -0.146 | -0.365 | 0.074 |
| Caring for others 1 or more hours |  |  |  |  |  |  |  |  | 0.215 | -0.056 | 0.486 |  | 0.217 | -0.043 | 0.477 |
| Doing housework 1 hour |  |  |  |  |  |  |  |  | -0.148 | -0.501 | 0.205 |  | 0.106 | -0.103 | 0.314 |
| Doing housework 2 or more hours |  |  |  |  |  |  |  |  | -0.188 | -0.571 | 0.196 |  | -0.014 | -0.234 | 0.206 |
| Financial situation |  |  |  |  |  |  |  |  | 0.117 | -0.060 | 0.295 |  | 0.136 | 0.039 | 0.232 |
| Working from home |  |  |  |  |  |  |  |  | 0.180 | -0.015 | 0.375 |  | 0.049 | -0.160 | 0.259 |
| Keyworker status |  |  |  |  |  |  |  |  | 0.106 | -0.126 | 0.338 |  | -0.021 | -0.221 | 0.180 |
| Dependent CYP in the household |  |  |  |  |  |  |  |  | -0.068 | -0.363 | 0.228 |  | -0.046 | -0.269 | 0.177 |
| **Effects on life satisfaction at age 50.5** |  |  |  |  |  |  |  |  |  |  |  |  |  |  |  |
| Working 1-8 hours |  |  |  |  |  |  |  |  | 0.333 | 0.102 | 0.565 |  | -0.193 | -0.490 | 0.104 |
| Working more than 8 hours |  |  |  |  |  |  |  |  | 0.247 | -0.008 | 0.501 |  | -0.302 | -0.631 | 0.027 |
| Volunteering 1 or more hours |  |  |  |  |  |  |  |  | 0.085 | -0.120 | 0.291 |  | 0.216 | -0.038 | 0.471 |
| Home-schooling 1 or more hours |  |  |  |  |  |  |  |  | -0.157 | -0.432 | 0.118 |  | -0.157 | -0.518 | 0.204 |
| Caring for children 1 or more hours |  |  |  |  |  |  |  |  | 0.116 | -0.057 | 0.290 |  | 0.064 | -0.186 | 0.314 |
| Caring for others 1 or more hours |  |  |  |  |  |  |  |  | -0.204 | -0.402 | -0.005 |  | 0.283 | -0.059 | 0.624 |
| Doing housework 1 hour |  |  |  |  |  |  |  |  | -0.160 | -0.428 | 0.108 |  | 0.376 | 0.100 | 0.651 |
| Doing housework 2 or more hours |  |  |  |  |  |  |  |  | -0.112 | -0.371 | 0.147 |  | 0.271 | -0.064 | 0.606 |
| Financial situation |  |  |  |  |  |  |  |  | 0.109 | 0.012 | 0.205 |  | 0.157 | 0.053 | 0.261 |
| Working from home |  |  |  |  |  |  |  |  | 0.375 | 0.200 | 0.550 |  | 0.216 | 0.002 | 0.430 |
| Keyworker status |  |  |  |  |  |  |  |  | 0.181 | -0.027 | 0.389 |  | -0.255 | -0.490 | -0.019 |
| Dependent CYP in the household |  |  |  |  |  |  |  |  | 0.027 | -0.239 | 0.292 |  | -0.026 | -0.298 | 0.246 |
| **Effects on life satisfaction at age 51** |  |  |  |  |  |  |  |  |  |  |  |  |  |  |  |
| Working 1-8 hours |  |  |  |  |  |  |  |  | 0.329 | 0.026 | 0.632 |  | 0.205 | -0.164 | 0.573 |
| Working more than 8 hours |  |  |  |  |  |  |  |  | 0.231 | -0.142 | 0.603 |  | 0.234 | -0.137 | 0.605 |
| Financial situation |  |  |  |  |  |  |  |  | 0.213 | 0.114 | 0.313 |  | 0.163 | 0.064 | 0.262 |
| Working from home |  |  |  |  |  |  |  |  | 0.081 | -0.145 | 0.306 |  | -0.019 | -0.269 | 0.230 |
| Keyworker status |  |  |  |  |  |  |  |  | -0.057 | -0.314 | 0.200 |  | 0.081 | -0.151 | 0.312 |
| Dependent CYP in the household |  |  |  |  |  |  |  |  | 0.119 | -0.200 | 0.437 |  | -0.147 | -0.423 | 0.128 |
| Phone interview | 1.264 | -1.150 | 3.678 |  | 0.115 | -0.809 | 1.040 |  | 1.645 | -0.610 | 3.900 |  | -0.027 | -1.266 | 1.213 |
|  |  |  |  |  |  |  |  |  |  |  |  |  |  |  |  |
| **Between-groups difference in coefficients **** | **Point estimate** | **95%**  **CI, LB** | **95% CI, UB** |  | **p-value** |  |  |  | **Point estimate** | **95%**  **CI, LB** | **95% CI, UB** |  | **p-value** |  |  |
| Mean intercept | 0.217 | 0.092 | 0.343 |  | 0.001 |  |  |  | 0.221 | 0.093 | 0.350 |  | 0.001 |  |  |
| Mean quadratic change, second segment | -0.021 | -0.031 | -0.011 |  | <0.001 |  |  |  | -0.027 | -0.044 | -0.010 |  | 0.002 |  |  |

*Note.* Models estimated using non-response weights and full information maximum likelihood under MLR estimation. Estimates in italics are constrained to be equal across groups. CI: confidence interval; CYP: children or young people; LB: lower bound; UB: upper bound. Effects of time use variables are estimated with no time spent in that activity as a reference category. * Adjusted for financial situation, working-from-home and keyworker status, presence in the household of dependent children or young people aged up to 16 years old, and interview mode. ** Between-groups difference in coefficients tested using Wald tests under the null hypothesis of estimate_women_ - estimate_men_ = 0.

## Appendix S11. Results from the multiple group latent growth curve models including lagged effects of the time-specific variables (n=6,766).

|  | **Women** | | |  | **Men** | | |
| --- | --- | --- | --- | --- | --- | --- | --- |
|  | **Point estimate** | **95% CI, LB** | **95% CI, UB** |  | **Point estimate** | **95% CI, LB** | **95% CI, UB** |
| **Means** |  |  |  |  |  |  |  |
| Intercept | 7.187 | 7.093 | 7.281 |  | 6.969 | 6.851 | 7.087 |
| Linear change, first segment | *0.041* | *0.026* | *0.057* |  | *0.041* | *0.026* | *0.057* |
| Quadratic change, first segment | *-0.002* | *-0.002* | *-0.001* |  | *-0.002* | *-0.002* | *-0.001* |
| Linear change, second segment | *0.133* | *-0.164* | *0.430* |  | *0.133* | *-0.164* | *0.430* |
| Quadratic change, second segment | -0.061 | -0.132 | 0.011 |  | -0.036 | -0.109 | 0.038 |
| **Variances** |  |  |  |  |  |  |  |
| Intercept | *1.712* | *1.475* | *1.949* |  | *1.712* | *1.475* | *1.949* |
| Linear change, first segment | *0.116* | *0.086* | *0.147* |  | *0.116* | *0.086* | *0.147* |
| Quadratic change, first segment | *0* |  |  |  | *0* |  |  |
| Linear change, second segment | *1.918* | *1.513* | *2.323* |  | *1.918* | *1.513* | *2.323* |
| Quadratic change, second segment | *0* |  |  |  | *0* |  |  |
| **Standardised covariances (correlations)** |  |  |  |  |  |  |  |
| Intercept x linear change, first segment | -0.349 | -0.477 | -0.221 |  | -0.324 | -0.453 | -0.194 |
| Intercept x linear change, second segment | -0.081 | -0.199 | 0.037 |  | -0.097 | -0.260 | 0.067 |
| Linear change first x second segment | -0.103 | -0.273 | 0.066 |  | -0.145 | -0.334 | 0.044 |
| **Standardised residual variances** |  |  |  |  |  |  |  |
| Age 26 | 0.554 | 0.498 | 0.610 |  | 0.550 | 0.489 | 0.610 |
| Age 30 | 0.563 | 0.516 | 0.609 |  | 0.514 | 0.455 | 0.574 |
| Age 34 | 0.545 | 0.499 | 0.590 |  | 0.479 | 0.419 | 0.539 |
| Age 42 | 0.543 | 0.482 | 0.604 |  | 0.503 | 0.449 | 0.557 |
| Age 46 | 0.393 | 0.319 | 0.467 |  | 0.379 | 0.301 | 0.457 |
| Age 50 | 0.400 | 0.336 | 0.464 |  | 0.269 | 0.220 | 0.318 |
| Age 50.5 | 0.278 | 0.237 | 0.318 |  | 0.281 | 0.222 | 0.339 |
| Age 51 | 0.339 | 0.292 | 0.386 |  | 0.268 | 0.218 | 0.318 |
| **Lagged effects on life satisfaction at age 50.5 (from age 50)** | |  |  |  |  |  |  |
| Working 1-8 hours | -0.057 | -0.288 | 0.175 |  | -0.163 | -0.435 | 0.109 |
| Working more than 8 hours | -0.101 | -0.384 | 0.181 |  | -0.095 | -0.376 | 0.187 |
| Volunteering 1 or more hours | 0.129 | -0.091 | 0.349 |  | 0.375 | 0.004 | 0.747 |
| Home-schooling 1 or more hours | -0.096 | -0.338 | 0.146 |  | 0.054 | -0.216 | 0.325 |
| Caring for children 1 or more hours | 0.223 | -0.015 | 0.461 |  | 0.090 | -0.212 | 0.393 |
| Caring for others 1 or more hours | -0.088 | -0.308 | 0.132 |  | 0.030 | -0.341 | 0.402 |
| Doing housework 1 hour | 0.146 | -0.131 | 0.423 |  | 0.068 | -0.183 | 0.318 |
| Doing housework 2 or more hours | 0.158 | -0.133 | 0.449 |  | 0.041 | -0.215 | 0.297 |
| Financial situation | 0.011 | -0.112 | 0.134 |  | -0.052 | -0.161 | 0.056 |
| Working from home | 0.034 | -0.146 | 0.214 |  | 0.128 | -0.079 | 0.336 |
| Keyworker status | 0.041 | -0.151 | 0.233 |  | -0.190 | -0.419 | 0.040 |
| Dependent CYP in the household |  |  |  |  |  |  |  |
| **Lagged effects on life satisfaction at age 51 (from age 50.5)** | |  |  |  |  |  |  |
| Working 1-8 hours | -0.280 | -0.537 | -0.023 |  | 0.189 | -0.156 | 0.534 |
| Working more than 8 hours | -0.252 | -0.537 | 0.033 |  | 0.289 | -0.060 | 0.638 |
| Volunteering 1 or more hours | 0.105 | -0.139 | 0.349 |  | -0.077 | -0.364 | 0.209 |
| Home-schooling 1 or more hours | 0.101 | -0.211 | 0.412 |  | 0.114 | -0.234 | 0.462 |
| Caring for children 1 or more hours | -0.067 | -0.268 | 0.133 |  | -0.020 | -0.266 | 0.226 |
| Caring for others 1 or more hours | 0.106 | -0.141 | 0.353 |  | -0.212 | -0.536 | 0.113 |
| Doing housework 1 hour | 0.211 | -0.188 | 0.611 |  | -0.266 | -0.572 | 0.039 |
| Doing housework 2 or more hours | 0.214 | -0.192 | 0.620 |  | -0.266 | -0.630 | 0.097 |
| Financial situation | 0.013 | -0.117 | 0.142 |  | -0.073 | -0.179 | 0.033 |
| Working from home | -0.210 | -0.400 | -0.019 |  | -0.171 | -0.355 | 0.012 |
| Keyworker status | -0.025 | -0.231 | 0.182 |  | 0.052 | -0.136 | 0.241 |
| Dependent CYP in the household | 0.090 | -0.171 | 0.351 |  | 0.049 | -0.199 | 0.297 |
| **Concurrent effects on life satisfaction at age 51** |  |  |  |  |  |  |  |
| Phone interview | 1.588 | -0.540 | 3.716 |  | 0.165 | -1.140 | 1.471 |
|  |  |  |  |  |  |  |  |
| **Between-groups difference in coefficients *** | **Point estimate** | **95%**  **CI, LB** | **95% CI, UB** |  | **p-value** |  |  |
| Mean intercept | 0.218 | 0.090 | 0.345 |  | 0.001 |  |  |
| Mean quadratic change, second segment | -0.025 | -0.039 | -0.011 |  | <0.001 |  |  |

*Note.* Models estimated using non-response weights and full information maximum likelihood under MLR estimation. Estimates in italics are constrained to be equal across groups. CI: confidence interval; CYP: children or young people; LB: lower bound; UB: upper bound. Effects of time use variables are estimated with no time spent in that activity as a reference category. * Between-groups difference in coefficients tested using Wald tests under the null hypothesis of estimate_women_ - estimate_men_ = 0.

## Appendix S12. Results from the multiple group latent growth curve models including treating high self-reported values of hours spent doing paid work (≥20h/day) as missing data (n=6,766).

|  | **Time use and mode effects only** | | | | | | |  | **Fully adjusted** | | | | | | |
| --- | --- | --- | --- | --- | --- | --- | --- | --- | --- | --- | --- | --- | --- | --- | --- |
|  | **Women** | | |  | **Men** | | |  | **Women** | | |  | **Men** | | |
|  | **Point estimate** | **95% CI, LB** | **95% CI, UB** |  | **Point estimate** | **95% CI, LB** | **95% CI, UB** |  | **Point estimate** | **95% CI, LB** | **95% CI, UB** |  | **Point estimate** | **95% CI, LB** | **95% CI, UB** |
| **Means** |  |  |  |  |  |  |  |  |  |  |  |  |  |  |  |
| Intercept | 7.191 | 7.096 | 7.285 |  | 6.969 | 6.851 | 7.088 |  | 7.188 | 7.093 | 7.283 |  | 6.967 | 6.848 | 7.085 |
| Linear change, first segment | *0.041* | *0.026* | *0.057* |  | *0.041* | *0.026* | *0.057* |  | *0.041* | *0.026* | *0.057* |  | *0.041* | *0.026* | *0.057* |
| Quadratic change, first segment | *-0.002* | *-0.002* | *-0.001* |  | *-0.002* | *-0.002* | *-0.001* |  | *-0.002* | *-0.002* | *-0.001* |  | *-0.002* | *-0.002* | *-0.001* |
| Linear change, second segment | *0.308* | *-0.062* | *0.679* |  | *0.308* | *-0.062* | *0.679* |  | *0.320* | *-0.049* | *0.689* |  | *0.320* | *-0.049* | *0.689* |
| Quadratic change, second segment | -0.103 | -0.178 | -0.029 |  | -0.083 | -0.167 | 0.001 |  | -0.107 | -0.182 | -0.032 |  | -0.080 | -0.162 | 0.002 |
| **Variances** |  |  |  |  |  |  |  |  |  |  |  |  |  |  |  |
| Intercept | *1.717* | *1.479* | *1.955* |  | *1.717* | *1.479* | *1.955* |  | *1.720* | *1.481* | *1.960* |  | *1.720* | *1.481* | *1.960* |
| Linear change, first segment | *0.116* | *0.086* | *0.147* |  | *0.116* | *0.086* | *0.147* |  | *0.116* | *0.086* | *0.146* |  | *0.116* | *0.086* | *0.146* |
| Quadratic change, first segment | *0* |  |  |  | *0* |  |  |  | *0* |  |  |  | *0* |  |  |
| Linear change, second segment | *1.842* | *1.423* | *2.261* |  | *1.842* | *1.423* | *2.261* |  | *1.801* | *1.428* | *2.174* |  | *1.801* | *1.428* | *2.174* |
| Quadratic change, second segment | *0* |  |  |  | *0* |  |  |  | *0* |  |  |  | *0* |  |  |
| **Standardised covariances (correlations)** | |  |  |  |  |  |  |  |  |  |  |  |  |  |  |
| Intercept x linear change, first segment | -0.349 | -0.476 | -0.221 |  | -0.325 | -0.455 | -0.195 |  | -0.347 | -0.475 | -0.218 |  | -0.323 | -0.453 | -0.192 |
| Intercept x linear change, second segment | -0.102 | -0.22 | 0.016 |  | -0.125 | -0.289 | 0.039 |  | -0.122 | -0.241 | -0.003 |  | -0.145 | -0.311 | 0.021 |
| Linear change first x second segment | -0.118 | -0.29 | 0.054 |  | -0.131 | -0.323 | 0.061 |  | -0.127 | -0.297 | 0.044 |  | -0.119 | -0.314 | 0.076 |
| **Standardised residual variances** |  |  |  |  |  |  |  |  |  |  |  |  |  |  |  |
| Age 26 | 0.554 | 0.497 | 0.610 |  | 0.547 | 0.488 | 0.606 |  | 0.554 | 0.498 | 0.610 |  | 0.547 | 0.488 | 0.605 |
| Age 30 | 0.561 | 0.515 | 0.607 |  | 0.513 | 0.454 | 0.573 |  | 0.561 | 0.515 | 0.607 |  | 0.512 | 0.452 | 0.572 |
| Age 34 | 0.545 | 0.499 | 0.590 |  | 0.479 | 0.420 | 0.539 |  | 0.542 | 0.496 | 0.588 |  | 0.479 | 0.419 | 0.539 |
| Age 42 | 0.542 | 0.482 | 0.603 |  | 0.503 | 0.448 | 0.557 |  | 0.542 | 0.482 | 0.603 |  | 0.500 | 0.446 | 0.555 |
| Age 46 | 0.390 | 0.317 | 0.464 |  | 0.381 | 0.302 | 0.459 |  | 0.391 | 0.318 | 0.464 |  | 0.382 | 0.306 | 0.459 |
| Age 50 | 0.410 | 0.340 | 0.481 |  | 0.269 | 0.221 | 0.317 |  | 0.410 | 0.348 | 0.471 |  | 0.270 | 0.222 | 0.318 |
| Age 50.5 | 0.277 | 0.232 | 0.321 |  | 0.293 | 0.232 | 0.353 |  | 0.281 | 0.239 | 0.324 |  | 0.288 | 0.231 | 0.345 |
| Age 51 | 0.336 | 0.288 | 0.384 |  | 0.258 | 0.203 | 0.314 |  | 0.322 | 0.277 | 0.367 |  | 0.255 | 0.207 | 0.302 |
| **Effects on life satisfaction at age 50** |  |  |  |  |  |  |  |  |  |  |  |  |  |  |  |
| Working 1-8 hours | 0.203 | -0.045 | 0.451 |  | 0.140 | -0.079 | 0.358 |  | 0.125 | -0.166 | 0.416 |  | 0.114 | -0.134 | 0.361 |
| Working more than 8 hours | 0.133 | -0.186 | 0.453 |  | -0.037 | -0.299 | 0.225 |  | 0.066 | -0.286 | 0.418 |  | -0.049 | -0.325 | 0.228 |
| Volunteering 1 or more hours | -0.191 | -0.447 | 0.065 |  | 0.093 | -0.247 | 0.432 |  | -0.173 | -0.421 | 0.076 |  | 0.088 | -0.243 | 0.418 |
| Home-schooling 1 or more hours | 0.136 | -0.114 | 0.387 |  | -0.030 | -0.256 | 0.195 |  | 0.238 | -0.063 | 0.538 |  | -0.068 | -0.298 | 0.162 |
| Caring for children 1 or more hours | -0.159 | -0.420 | 0.103 |  | -0.134 | -0.332 | 0.065 |  | -0.069 | -0.336 | 0.197 |  | -0.146 | -0.365 | 0.073 |
| Caring for others 1 or more hours | 0.242 | -0.037 | 0.521 |  | 0.197 | -0.059 | 0.453 |  | 0.216 | -0.055 | 0.487 |  | 0.212 | -0.049 | 0.473 |
| Doing housework 1 hour | -0.112 | -0.461 | 0.237 |  | 0.109 | -0.097 | 0.315 |  | -0.139 | -0.492 | 0.213 |  | 0.102 | -0.107 | 0.310 |
| Doing housework 2 or more hours | -0.140 | -0.507 | 0.227 |  | -0.013 | -0.246 | 0.220 |  | -0.179 | -0.562 | 0.204 |  | -0.029 | -0.252 | 0.194 |
| Financial situation |  |  |  |  |  |  |  |  | 0.120 | -0.058 | 0.298 |  | 0.136 | 0.039 | 0.233 |
| Working from home |  |  |  |  |  |  |  |  | 0.199 | 0.006 | 0.393 |  | 0.042 | -0.168 | 0.252 |
| Keyworker status |  |  |  |  |  |  |  |  | 0.132 | -0.099 | 0.362 |  | -0.039 | -0.239 | 0.162 |
| Dependent CYP in household |  |  |  |  |  |  |  |  | -0.064 | -0.360 | 0.232 |  | -0.048 | -0.270 | 0.175 |
| **Effects on life satisfaction at age 50.5** |  |  |  |  |  |  |  |  |  |  |  |  |  |  |  |
| Working 1-8 hours | 0.451 | 0.216 | 0.686 |  | -0.141 | -0.438 | 0.156 |  | 0.286 | 0.049 | 0.523 |  | -0.233 | -0.535 | 0.069 |
| Working more than 8 hours | 0.353 | 0.075 | 0.631 |  | -0.297 | -0.644 | 0.050 |  | 0.195 | -0.087 | 0.477 |  | -0.331 | -0.674 | 0.011 |
| Volunteering 1 or more hours | 0.088 | -0.117 | 0.293 |  | 0.268 | 0.011 | 0.526 |  | 0.084 | -0.121 | 0.289 |  | 0.216 | -0.036 | 0.468 |
| Home-schooling 1 or more hours | -0.122 | -0.393 | 0.149 |  | -0.102 | -0.461 | 0.258 |  | -0.158 | -0.433 | 0.117 |  | -0.151 | -0.506 | 0.204 |
| Caring for children 1 or more hours | 0.125 | -0.031 | 0.281 |  | 0.118 | -0.092 | 0.328 |  | 0.116 | -0.058 | 0.289 |  | 0.064 | -0.185 | 0.313 |
| Caring for others 1 or more hours | -0.187 | -0.381 | 0.008 |  | 0.215 | -0.108 | 0.537 |  | -0.206 | -0.405 | -0.007 |  | 0.283 | -0.056 | 0.622 |
| Doing housework 1 hour | -0.110 | -0.382 | 0.161 |  | 0.382 | 0.104 | 0.659 |  | -0.136 | -0.405 | 0.134 |  | 0.389 | 0.112 | 0.666 |
| Doing housework 2 or more hours | -0.072 | -0.332 | 0.188 |  | 0.230 | -0.103 | 0.563 |  | -0.089 | -0.348 | 0.169 |  | 0.262 | -0.070 | 0.594 |
| Financial situation |  |  |  |  |  |  |  |  | 0.107 | 0.010 | 0.203 |  | 0.161 | 0.057 | 0.265 |
| Working from home |  |  |  |  |  |  |  |  | 0.381 | 0.206 | 0.557 |  | 0.229 | 0.015 | 0.442 |
| Keyworker status |  |  |  |  |  |  |  |  | 0.190 | -0.020 | 0.400 |  | -0.242 | -0.479 | -0.005 |
| Dependent CYP in household |  |  |  |  |  |  |  |  | 0.026 | -0.240 | 0.292 |  | -0.026 | -0.297 | 0.245 |
| **Effects on life satisfaction at age 51** |  |  |  |  |  |  |  |  |  |  |  |  |  |  |  |
| Working 1-8 hours | 0.274 | 0.040 | 0.509 |  | 0.293 | -0.038 | 0.624 |  | 0.316 | 0.010 | 0.622 |  | 0.194 | -0.175 | 0.563 |
| Working more than 8 hours | 0.141 | -0.177 | 0.459 |  | 0.329 | -0.007 | 0.664 |  | 0.201 | -0.180 | 0.581 |  | 0.237 | -0.135 | 0.608 |
| Financial situation |  |  |  |  |  |  |  |  | 0.214 | 0.114 | 0.314 |  | 0.162 | 0.063 | 0.261 |
| Working from home |  |  |  |  |  |  |  |  | 0.086 | -0.139 | 0.312 |  | -0.017 | -0.266 | 0.232 |
| Keyworker status |  |  |  |  |  |  |  |  | -0.052 | -0.309 | 0.205 |  | 0.086 | -0.146 | 0.317 |
| Dependent CYP in household |  |  |  |  |  |  |  |  | 0.118 | -0.201 | 0.438 |  | -0.150 | -0.425 | 0.126 |
| Phone interview | 1.781 | -0.394 | 3.956 |  | 0.097 | -2.272 | 2.466 |  | 1.648 | -0.563 | 3.859 |  | -0.047 | -1.244 | 1.151 |
|  |  |  |  |  |  |  |  |  |  |  |  |  |  |  |  |
| **Between-groups difference in coefficients *** | **Point estimate** | **95% CI, LB** | **95% CI, UB** |  | **p-value** |  |  |  | **Point estimate** | **95% CI, LB** | **95% CI, UB** |  | **p-value** |  |  |
| Mean intercept | 0.221 | 0.093 | 0.350 |  | 0.001 |  |  |  | 0.221 | 0.093 | 0.350 |  | 0.001 |  |  |
| Mean quadratic change, second segment | -0.021 | -0.036 | -0.003 |  | 0.023 |  |  |  | -0.027 | -0.044 | -0.010 |  | 0.002 |  |  |

*Note.* Models estimated using non-response weights and full information maximum likelihood under MLR estimation. Estimates in italics are constrained to be equal across groups. CI: confidence interval; CYP: children or young people; LB: lower bound; UB: upper bound. Effects of time use variables are estimated with no time spent in that activity as a reference category. * Between-groups difference in coefficients tested using Wald tests under the null hypothesis of estimate_women_ - estimate_men_ = 0.
